# RBM47 regulates intestinal injury and tumorigenesis by modifying proliferation, oxidative response, and inflammatory pathways

**DOI:** 10.1172/jci.insight.161118

**Published:** 2023-05-08

**Authors:** Saeed Soleymanjahi, Valerie Blanc, Elizabeth A. Molitor, David M. Alvarado, Yan Xie, Vered Gazit, Jeffrey W. Brown, Kathleen Byrnes, Ta-Chiang Liu, Jason C. Mills, Matthew A. Ciorba, Deborah C. Rubin, Nicholas O. Davidson

**Affiliations:** 1 Division of Gastroenterology, Department of Medicine;; 2 Department of Developmental Biology; and; 3 Department of Pathology & Immunology, Washington University School of Medicine in St. Louis, St. Louis, Missouri, USA.

**Keywords:** Gastroenterology, Inflammation, Colorectal cancer, RNA processing

## Abstract

RNA-binding protein 47 (RBM47) is required for embryonic endoderm development, but a role in adult intestine is unknown. We studied intestine-specific *Rbm47*-knockout mice (*Rbm47-IKO*) following intestinal injury and made crosses into *Apc^Min/+^* mice to examine alterations in intestinal proliferation, response to injury, and tumorigenesis. We also interrogated human colorectal polyps and colon carcinoma tissue. *Rbm47-IKO* mice exhibited increased proliferation and abnormal villus morphology and cellularity, with corresponding changes in *Rbm47-IKO* organoids. *Rbm47-IKO* mice adapted to radiation injury and were protected against chemical-induced colitis, with *Rbm47-IKO* intestine showing upregulation of antioxidant and Wnt signaling pathways as well as stem cell and developmental genes. Furthermore, *Rbm47-IKO* mice were protected against colitis-associated cancer. By contrast, aged *Rbm47-IKO* mice developed spontaneous polyposis, and *Rbm47-IKO* *Apc^Min/+^* mice manifested an increased intestinal polyp burden. *RBM47* mRNA was decreased in human colorectal cancer versus paired normal tissue, along with alternative splicing of tight junction protein 1 mRNA. Public databases revealed stage-specific reduction in RBM47 expression in colorectal cancer associated independently with decreased overall survival. These findings implicate RBM47 as a cell-intrinsic modifier of intestinal growth, inflammatory, and tumorigenic pathways.

## Graphical abstract

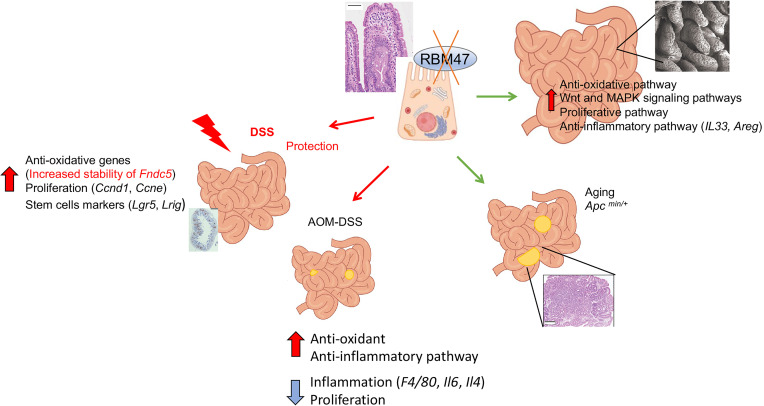

## Introduction

RNA-binding proteins (RBPs) orchestrate a range of developmental events in embryogenesis and induction of pluripotency through effects including mRNA processing and subcellular localization as well as mRNA translation and degradation (1). The human genome encodes more than 1,500 RBPs, of which the largest subset contains at least 1 copy of a canonical RNA recognition motif (2). Among this family of RNA recognition motif–containing RBPs, RNA-binding protein 47 (RBM47) was identified as a differentially expressed gene in vertebrate foregut endoderm (3). Studies in zebrafish and mice demonstrated that *Rbm47* is required for embryonic development, with roles in zebrafish head and mouse foregut development (4, 5). In particular, RBM47 is abundantly expressed in early embryonic (E8.5) foregut and in adult mouse tissues, including small intestinal epithelium, liver, and pancreas (5).

A role for RBM47 in intestinal homeostasis emerged following conditional expression of a hypomorphic *Rbm47* allele, which revealed defective fetal intestinal development with growth retardation and runting of small numbers of viable offspring (5, 6). In addition, those studies revealed an unanticipated role in RNA editing of apolipoprotein B, suggesting a new function for RBM47 in posttranscriptional gene regulation and in mammalian intestinal lipid metabolism (6). To circumvent the embryonic lethality encountered with earlier approaches, we generated intestine-specific Rbm47-null mice (*Rbm47-IKO*) and demonstrated a hierarchy of APOBEC1-dependent C-to-U RNA editing target requirements for the canonical RBPs, namely Apobec1 complementation factor and RBM47, whose effects and roles exhibit striking tissue specificity (7, 8). Those studies, however, left unanswered the role and functions of RBM47 in adult intestine.

Mammalian small intestine is a dynamic tissue whose spatial organization, as well as growth, development, and proliferation, are all highly regulated (9). In this context, studies have implicated a role for RBPs in both health and disease (10), including in the mammalian intestine (11). More specifically, studies have demonstrated a role for RBM47 in cell fate decisions, including in murine embryonic stem cells, which identified *Nanog* as a direct target (12), with other studies showing that RBM47 modulates developmental signaling via pathways including *Wnt8a*, *Epcam*, and *Chordin* (4, 13). Work has also demonstrated that RBM47 influences cell fate through p53/p21 signaling pathways (14) and via AXIN1 (15). Those latter studies, reflecting work in cancer cell lines, support an emerging consensus that RBM47 likely functions as a tumor suppressor in both breast and colon cancer (14), with a role in cancer metastasis (16).

The current studies explore in depth a role for intestinal RBM47 in small intestinal epithelial homeostasis and the response to injury. We show that *Rbm47-IKO* mice exhibited abnormal villus morphology, with increased proliferation and augmented adaptation following injury. Those adaptations were mediated through upregulation of stem cell proliferative pathways and altered WNT and MAPK signaling, along with alterations in glutathione turnover and oxidation pathways. In addition, we found reduced expression of RBM47 in samples of human colorectal cancer compared with paired normal tissue, and databases revealed decreased overall survival with low RBM47 expression. These findings implicate RBM47 as a cell-intrinsic epithelial modifier of intestinal development and growth pathways as well as in tumorigenesis.

## Results

### Rbm47-IKO mice exhibit increased intestinal proliferation and altered morphology.

**Figure 1:**
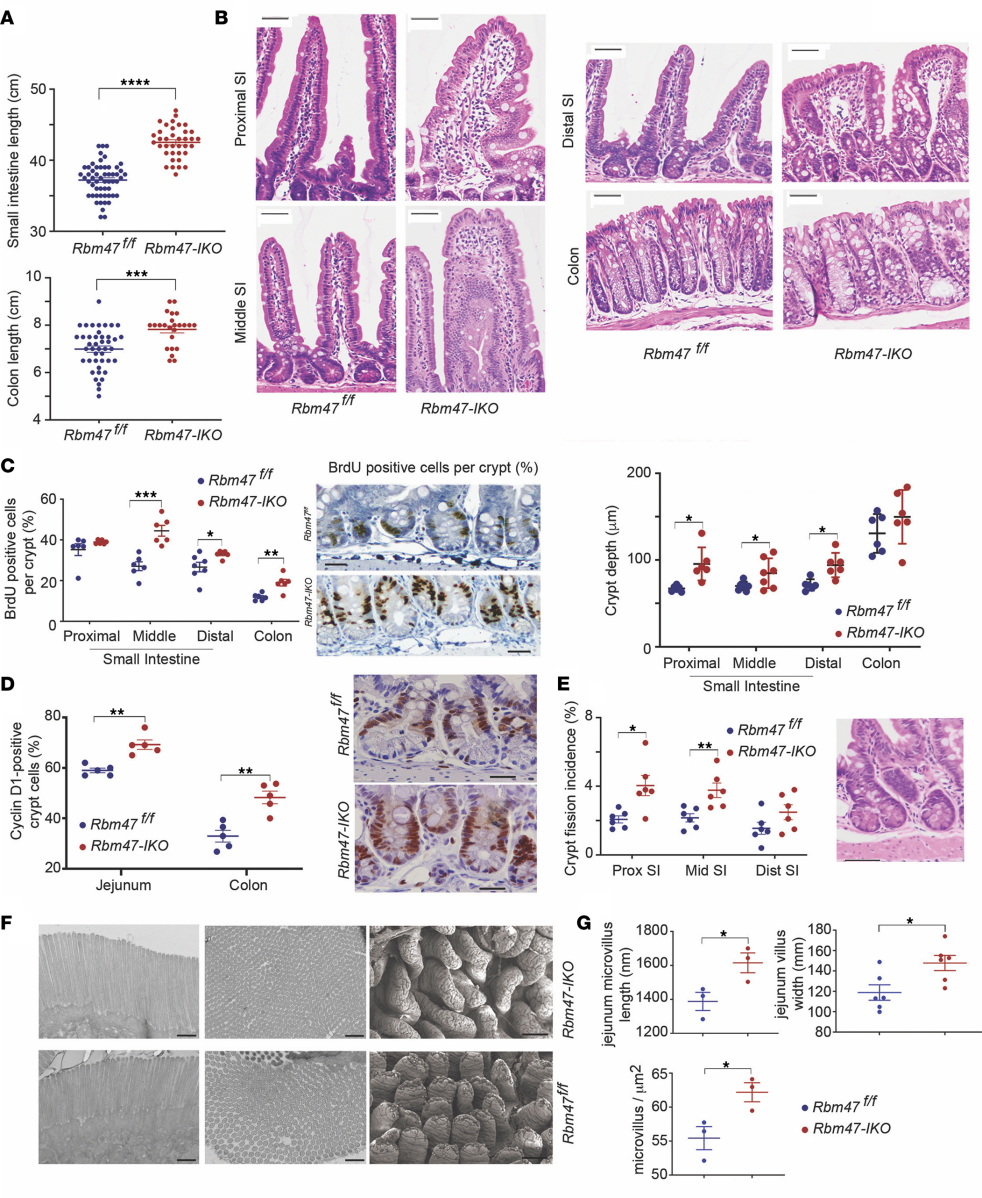
Constitutive intestinal *Rbm47* deletion enhances the proliferation capacity and anatomical development of intestinal epithelium in young (8–14 weeks) mice. (**A**) Small intestine (top) and colon (bottom) length in *Rbm47^fl/fl^* and *Rbm47-IKO* mice (unpaired *t* test, *n* = 55 *fl/fl*, 37 *IKO* for small intestine and 43 *fl/fl*, 24 *IKO* for colon). (**B**) Top: regional sections of small intestine and colon from *Rbm47^fl/fl^* and *Rbm47-IKO* mice (scale bar: 50 μm). Bottom: Crypt depth in *Rbm47^fl/fl^* and *Rbm47-IKO* mice (unpaired *t* test, *n* = 6/genotype, 30–40 crypts with longitudinal cross-sectional view per mouse). (**C**) Left: Regional BrdU-positive abundance (% per total crypt cells) in small intestine and colon from *Rbm47^fl/fl^* and *Rbm47-IKO* mice (unpaired *t* test, *n* = 6/genotype, 20–30 crypts with complete longitudinal cross-sectional view per mouse). Right: BrdU-stained sections of middle small intestine crypts (scale bar: 25 μm). (**D**) Left: Cyclin D1–positive abundance (% per total crypt cells) in jejunum and colon from young *Rbm47^fl/fl^* and *Rbm47-IKO* mice (unpaired *t* test, *n* = 5/genotype, 20–30 crypts with longitudinal cross-sectional view per mouse); Right: Cyclin D1-stained sections of middle small intestine crypts (scale bar: 25 μm). (**E**) Left: regional crypt fission in small intestine from *Rbm47^fl/fl^* and *Rbm47-IKO* mice (unpaired *t* test, *n* = 6/genotype, 200 crypts with longitudinal cross-sectional view per mouse per section). Right: H&E-stained view of crypt fission (scale bar: 50 μm). (**F**) Transmission electron microscopic sections exhibiting length (left) and cross-sectional density (middle) of microvilli (scale bar: 500 nm) and scanning electron microscopic sections of villi (right, scale bar: 100 μm) in middle small intestine of young mice. (**G**) Left: Length (top) and density (bottom) of microvilli in middle small intestine from *Rbm47^fl/fl^* and *Rbm47-IKO* mice (unpaired *t* test, *n* = 3/genotype, 75–100 microvilli per mouse). Right: middle small intestine villi width in *Rbm47^fl/fl^* and *Rbm47-IKO* mice (unpaired *t* test, *n* = 6/genotype, 60–80 villi per mouse). Data are presented as mean ± SEM. *P < 0.05, **P < 0.01, ***P < 0.001, and ****P < 0.0001.

Constitutive intestinal *Rbm47* deletion revealed negligible *Rbm47* mRNA and protein expression (Supplemental Figure 1; supplemental material available online with this article; https://doi.org/10.1172/jci.insight.161118DS1), with longer small intestine and colon (Figure 1A), deeper crypts, and abnormally shaped, wider villi (Figure 1B and Supplemental Figure 1), with no change in body weight (Supplemental Figure 1). We observed increased BrdU-positive (Figure 1C) and cyclin D1–positive (Figure 1D) intestinal and colonic crypt cells and increased crypt fission (Figure 1E) in *Rbm47-IKO* jejunum. Transmission electron microscopy revealed longer and more densely distributed microvilli, and scanning electron microscopy recapitulated light microscopic findings of abnormally shaped, wider villi in *Rbm47-IKO* jejunum (Figure 1, F and G, and Supplemental Video 1).

We observed that adult mice with inducible (ERT2-Cre) *Rbm47* deletion exhibited similarly abnormally shaped and wider villi, deeper crypts, and numerous crypt fissions (Supplemental Figure 1, E and F).

**Figure 2:**
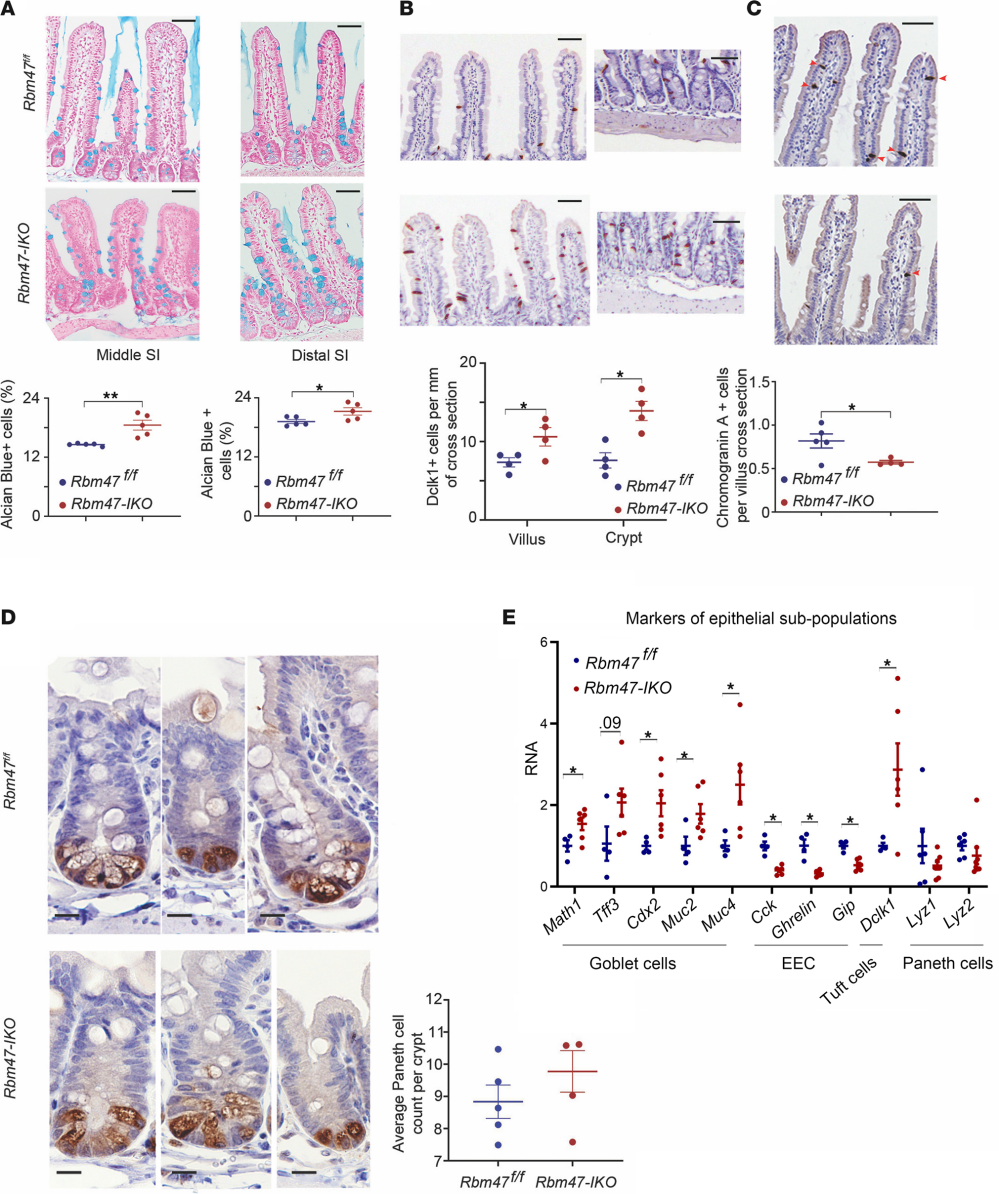
Constitutive intestinal *Rbm47* deletion alters abundance of differentiated cell subpopulations in intestinal epithelium of young (8–14 weeks) mice. (**A**) Top: Alcian blue–stained sections of middle (left) and distal (right) small intestine in *Rbm47^fl/fl^* and *Rbm47-IKO* mice (scale bar: 25 μm). Bottom: goblet cell abundance (% per total cells) in villi and crypts of middle (left graph) and distal (right graph) small intestine from *Rbm47^fl/fl^* and *Rbm47-IKO* mice (unpaired *t* test, *n* = 5/genotype, 30–40 villi and crypts with complete longitudinal cross-sectional view per mouse). (**B**) Top: Dclk1-stained sections of middle small intestine villi (left) and crypts (right) to demonstrate tuft cells in *Rbm47^fl/fl^* and *Rbm47-IKO* mice (scale bar: 25 μm). Bottom: tuft cell abundance (cell count/mm of border) in villi and crypts of middle small intestine from *Rbm47^fl/fl^* and *Rbm47-IKO* mice (unpaired *t* test, *n* = 4/genotype, 30–40 villi and crypts with complete longitudinal cross-sectional view per mouse). (**C**) Top: Chromogranin A–stained sections of middle small intestine villus to demonstrate enteroendocrine cells (EEC) (red arrowheads) in *Rbm47^fl/fl^* and *Rbm47-IKO* mice (scale bar: 25 μm). Bottom: EEC abundance (cell count per longitudinal cross-section of villus) in middle small intestine from *Rbm47^fl/fl^* and *Rbm47-IKO* mice (unpaired *t* test, *n* = 5 *fl/fl* and 4 *IKO*, 30–40 villi with complete longitudinal cross-sectional view per mouse). (**D**) Left: Lysozyme-stained sections of middle small intestine crypts to demonstrate Paneth cells in *Rbm47^fl/fl^* and *Rbm47-IKO* mice (scale bar: 20 μm). Right: Paneth cell abundance (cell count per crypt) in middle small intestine from *Rbm47^fl/fl^* and *Rbm47-IKO* mice (unpaired *t* test, *n* = 5/genotype, 35–40 crypts with complete longitudinal cross-sectional view per mouse). (**E**) qPCR evaluation of different epithelial subpopulation cell markers in middle small intestine from young *Rbm47^fl/fl^* and *Rbm47-IKO* mice (unpaired *t* test, *n* = 4 *fl/fl*, 6 *IKO*). Data are presented as mean ± SEM; *P < 0.05, and **P < 0.01.

We next examined the distribution of terminally differentiated intestinal cell subpopulations. *Rbm47-IKO* mice exhibited increased abundance of small intestinal goblet cells (Figure 2A) and tuft cells (Figure 2B), with reduced abundance of enteroendocrine cells (Figure 2C) and comparable abundance of Paneth cells (Figure 2D). Colon goblet cell abundance was unchanged by genotype (Supplemental Figure 2), with no change in mucus layer thickness or intestinal permeability as determined by FITC-labeled dextran (FD4) administration (Supplemental Figure 2). We further examined cell markers/genes related to different cell subpopulations, which revealed higher expression in *Rbm47-IKO* intestine of goblet cell–related genes (*Math1*, *Tff3*, *Cdx2*, *Muc2*, *Muc4*), lower expression in *Rbm47-IKO* intestine of enteroendocrine cell hormonal genes (*Cck*, *Ghrl*, *Gip*), higher expression in *Rbm47-IKO* intestine of *Dclk1* in *Rbm47-IKO* mice, and no change in expression of *Lys1*, *2* by genotype (Figure 2E). These findings collectively suggest that deletion of *Rbm47* in mouse intestine results in increased proliferative capacity and intestinal elongation, as well as selectively altered cell differentiation. Immunohistochemical staining for RBM47 revealed expression predominantly in differentiated enterocytes compared with stem cells and undifferentiated amplifying cells in *Rbm47*-floxed mice along with homogeneous loss of RBM47 expression in villi and crypts of *Rbm47-IKO* mouse intestine and colon (Supplemental Figure 1D). In support of these observations, single-cell RNA-Seq (scRNA-Seq) data from mouse intestine (17) revealed relatively decreased *Rbm47* mRNA expression in stem cells, Paneth cells, and transitional amplifying cells compared with differentiated enterocytes (Supplemental Figure 3).

### Isolated stem cell–derived enteroids and colonoids from Rbm47-IKO mice exhibit increased viability and proliferation.

**Figure 3:**
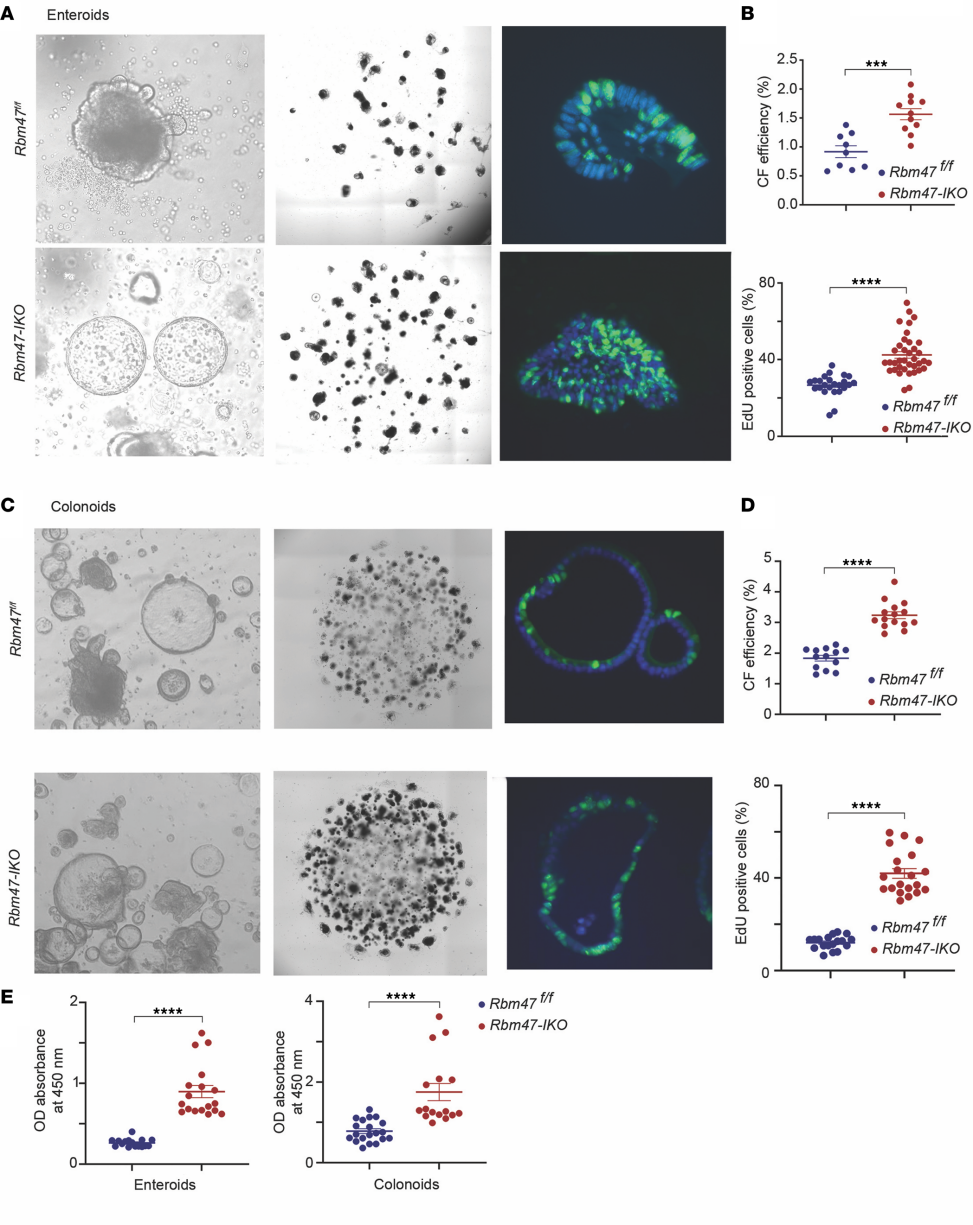
Constitutive intestinal *Rbm47* deletion increases cell viability and proliferation in young (8–14 weeks) mice’s stem cell–derived enteroids and colonoids. (**A**) Left: inverse light microscopic view of *Rbm47^fl/fl^* and *Rbm47-IKO* mice’s middle small intestine stem cell–derived organoids. Middle: Cytation 5 scanner view of colony-forming assay (CFA) using middle small intestine stem cell–derived organoids. Right: fluorescence microscopic images of EdU-stained (shown in green) middle small intestine stem cell–derived organoids. Hoechst (blue) was used for nuclear staining. (**B**) CFA (top) and abundance of EdU-positive cells (% of total cells per organoid) (bottom) in *Rbm47^fl/fl^* and *Rbm47-IKO* mice’s middle small intestine stem cell–derived organoids (mean ± SEM, unpaired *t* test, 9 *fl/fl* and 11 *IKO* experimental replicates for CFA; 24 *fl/fl* and 37 *IKO* organoids for EdU assay); ***P < 0.001; ****P < 0.0001. (**C**) Left: inverse light microscopic view of *Rbm47^fl/fl^* and *Rbm47-IKO* mice stem cell–derived colonoids. Middle: Cytation 5 scanner view of CFA using the colonoids. Right: fluorescence microscopic images of EdU-stained (green) colonoids. (**D**) CFA (top) and abundance of EdU-positive cells (% of total cells per organoid) (bottom) in *Rbm47^fl/fl^* and *Rbm47-IKO* mice’s stem cell–derived colonoids (mean ± SEM, unpaired *t* test, 13 *fl/fl* and 15 *IKO* experimental replicates for CFA; 18 *fl/fl* and 20 *IKO* organoids for EdU assay); ****P < 0.0001. (**E**) Cell viability assay using cell counting kit-8 method in *Rbm47^fl/fl^* and *Rbm47-IKO* mice’s stem cell–derived enteroids (left) and colonoids (right) (mean ± SEM, unpaired *t* test; *n* = 16 *fl/fl* and 18 *IKO* experimental replicates for enteroids; *n* = 20 *fl/fl* and 16 *IKO* experimental replicates for colonoids); ****P < 0.0001.

Based on our observations of enhanced proliferation and upregulation of cell proliferation genes in *Rbm47-IKO* intestine, we examined cell viability and growth capacity in isolated intestine and colon stem cell–derived organoids. We observed that both enteroids (Figure 3, A and B) and colonoids (Figure 3, C and D) derived from *Rbm47-IKO* isolated stem cells exhibited increased colony-forming efficiency, EdU-positive cell abundance, and cell viability (Figure 3E). These results suggest that the enhanced intestinal proliferation and growth observed in *Rbm47-IKO* mice reflects epithelial, cell-intrinsic adaptations including greater proliferative capacity and viability of stem cell–derived organoids. Furthermore, the cystic growth pattern observed is consistent with reports suggesting high WNT tone (18, 19) contributes to the increased intestinal proliferation observed in *Rbm47-IKO* enteroids.

### Transcriptome-wide analysis of Rbm47-IKO small intestine reveals upregulation of genes involved in development and morphogenesis, proliferation, and antioxidation response.

**Figure 4:**
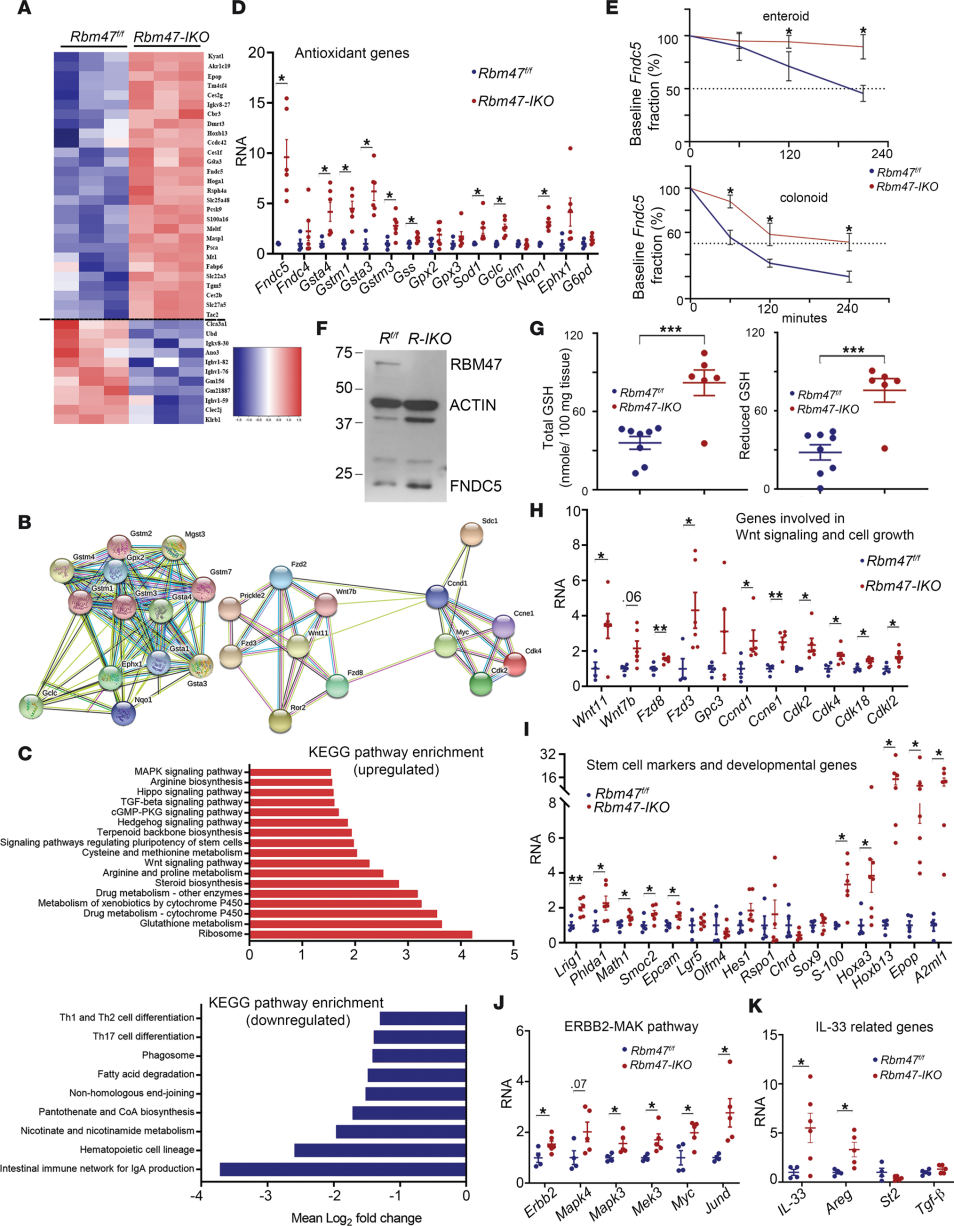
Differentially expressed genes in small intestine from young (8–14 weeks) *Rbm47-IKO* mice. (**A**) Heatmap illustration of top upregulated and downregulated genes in *Rbm47-IKO* versus *Rbm47^fl/fl^* mice. (**B**) STRING analysis of upregulated genes related to oxidative response (left) and WNT signaling (right). (**C**) Enriched Kyoto Encyclopedia of Genes and Genomes (KEGG) pathways in upregulated (top) and downregulated (bottom) genes. (**D**) qPCR evaluation of differentially upregulated genes involved in antioxidative response and glutathione metabolism (mean ± SEM, unpaired *t* test, *n* = 4 *fl/fl* and 6 *IKO*); *P < 0.05. (**E**) Relative *Fndc5* mRNA abundance at successive time points after actinomycin D treatment as a fraction of baseline *Fndc5* in enteroids (top) and colonoids (bottom) (mean ± SEM, unpaired *t* test, *n* = 3 independent experiments per genotype/time point); *P < 0.05. (**F**) Abundance of RBM47, ACTIN, and FNDC5 proteins in middle small intestine mucosa from *Rbm47^fl/fl^* and *Rbm47-IKO* mice (each lane represents a pool of 3 separate extracts per genotype). (**G**) Tissue content of total (left) and reduced (right) glutathione in middle small intestine from young mice (mean ± SEM, unpaired *t* test, *n* = 8 *fl/fl* and 6 *IKO*). ***P < 0.001. (**H**–**K**) qPCR evaluation of differentially upregulated genes involved in WNT signaling and cell proliferation (**H**), stem cell markers and developmental genes (**I**), genes involved in ERBB2/MAPK pathway (**J**), and IL-33–related genes (**K**) (mean ± SEM, unpaired *t* test, *n* = 4 *fl/fl* and 6 *IKO*); *P < 0.05, **P < 0.01.

RBM47 is an RBP regulating stability, alternative splicing, and translation of target mRNAs (4, 12, 14, 20). RNA-Seq from jejunal mucosa (Figure 4A) revealed *Epop*, *Gstm*, and *Fndc5* to be among highly upregulated genes in *Rbm47-IKO* mice (Figure 4A). STRING network and pathway enrichment analyses revealed alterations in functional pathways including glutathione metabolism, pathways regulating pluripotency of stem cells, cell proliferation pathways (e.g., *Wnt* and *Mapk* signaling), and innate and adaptive immunity and DNA repair pathways (Figure 4, B and C, and Supplemental Figure 4). We also aligned features (specifically AUUUA and polyU tracts) in the 3′ untranslated region of intestinal RNAs whose expression was changed with *Rbm47* deletion (Supplemental Figure 3). Quantitative PCR (qPCR) validated upregulation of mRNAs of interest related to glutathione metabolism and antioxidative response (e.g., fibronectin type III domain-containing protein 5 [*Fndc5*], different glutathione transferases, *Nqo1*, and *Sod1*), Wnt signaling (e.g., *Wnt11*, *Wnt7b*, *Fzd8*, *Fzd3*, *Ccnd1*), *ERBB2*/*MAPK* pathway (e.g., *Erbb2*, *Mapk*s, *Mek3*, *Myc*), intestinal stem cell markers (e.g., *Lrig1*, *Phlda1*, *Atoh1*, *Smoc2*), developmental genes (e.g., *Hoxa3*, *Hoxb13*, *Epop*, *A2ml1*), and IL-33–related genes (Figure 4, D and H–K).

In keeping with the upregulation of *Fndc5* mRNA, we observed increased FNDC5 protein abundance in *Rbm47-IKO* mice (Figure 4F). We further explored potential mechanisms underlying the upregulation of *Fndc5* mRNA and FNDC5 protein and observed increased *Fndc5* mRNA stability in enteroids and colonoids derived from *Rbm47-IKO* mice compared with control (i.e., floxed) mice (Figure 4E) and a shift toward increased polysomal distribution of *Fndc5* mRNA in *Rbm47-IKO* mice (Supplemental Figure 5) compared with control mice. Upregulation of genes involved in antioxidation response and glutathione metabolism was confirmed by approximately 2-fold higher tissue content of total and reduced glutathione in jejunum of *Rbm47-IKO* mice (Figure 4G). Taken together, these results demonstrate that intestinal epithelial RBM47 modifies expression of genes involved in cell proliferation, organogenesis, stem cell pluripotency, and antioxidative response pathways.

### Rbm47-IKO mice exhibit enhanced intestinal adaptation after whole-body γ-irradiation.

**Figure 5:**
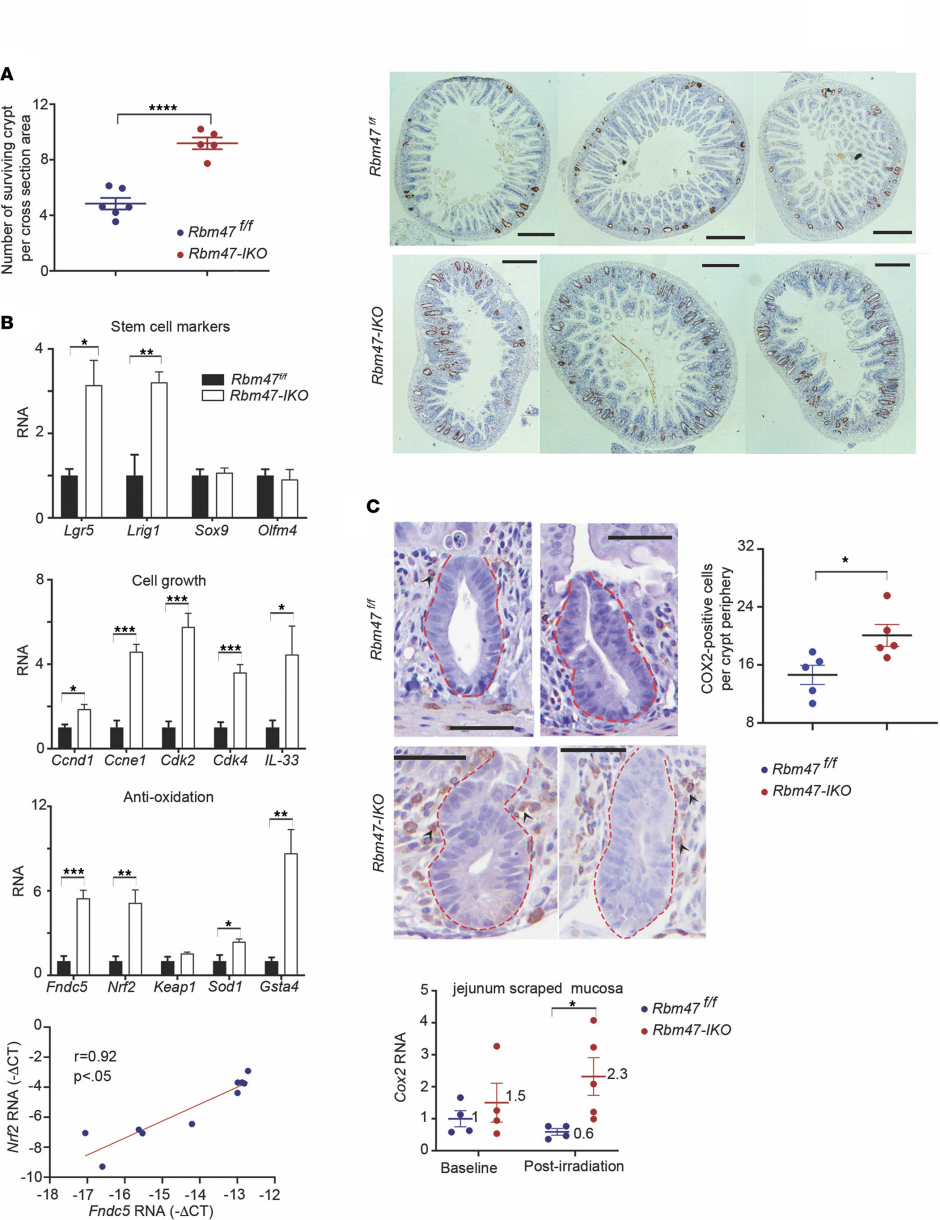
Constitutive intestinal *Rbm47* deletion promotes recovery of small intestine after 12 Gy whole-body irradiation. (**A**) Left: number of surviving crypts, determined using modified microcolony assay, per middle small intestine cross-sectional area in *Rbm47^fl/fl^* and *Rbm47-IKO* mice, 84 hours after whole-body irradiation (mean ± SEM, unpaired *t* test, *n* = 5/genotype, 6–8 cross sections per mouse). Right: BrdU-stained cross sections of middle small intestine from *Rbm47^fl/fl^* and *Rbm47-IKO* mice after irradiation (scale bar: 400 μm). (**B**) qPCR evaluation of stem cell markers, cell growth genes, and antioxidative genes’ expressions as well as the association between *Fndc5* and *Nrf2* expressions in middle small intestine from irradiated *Rbm47^fl/fl^* and *Rbm47-IKO* mice (mean ± SEM and unpaired *t* test for gene expressions, Pearson’s *r* test for correlation, *n* = 5/genotype); *P < 0.05, **P < 0.01, ***P < 0.001. (**C**) Top left: COX2 immunohistochemical staining of middle small intestine from irradiated *Rbm47^fl/fl^* and *Rbm47-IKO* mice (scale bar: 50 μm). Pericryptal COX2-positive cells are marked by black arrowheads. Top right: number of pericryptal COX2-positive cells/mm crypt periphery (mean ± SEM, unpaired *t* test, *n* = 5/genotype, 20–30 crypts with complete longitudinal cross-sectional view per mouse); *P < 0.05. Bottom: *Cox2* mRNA expression in middle small intestine from *Rbm47^fl/fl^* and *Rbm47-IKO* mice before and after irradiation (mean ± SEM, unpaired *t* test, *n* = 4/genotype for each assay); *P < 0.05.

We next asked if *Rbm47-IKO* mice exhibit protection against intestinal injury. We examined the response to whole-body γ-irradiation injury using a microcolony assay to evaluate surviving crypts 84 hours after irradiation (21, 22). We observed an approximately 2-fold higher number of surviving crypts in *Rbm47-IKO* jejunum after irradiation (Figure 5A), with 3-fold upregulation of stem cell markers *Lgr5* and *Lrig1*, 2- to 6-fold upregulation of cell proliferation genes (*Ccne1*, *Ccnd1*), and 5-fold upregulation of antioxidative genes *Fndc5* and *Nrf2* in jejunal mucosa of irradiated *Rbm47-IKO* mice (Figure 5B). We also found that jejunal *Fndc5* expression strongly (*r* = 0.92) correlated with *Nrf2* expression (Figure 5B) after irradiation, which is relevant because *Fndc5* was among the top upregulated genes in *Rbm47-IKO* jejunum and because of other work showing that *Fndc5* regulates expression of *Nrf2*, a key regulator of antioxidation response genes (23–26). We considered the possibility that changes in apoptosis might also contribute to the phenotypes observed, but crypt cell apoptosis measured 6 hours after radiation injury was comparable by genotype (Supplemental Figure 6A). We also observed a 4-fold increase in *Cox2* mRNA expression with increased pericryptal COX2-positive cells in *Rbm47-IKO* mice (Figure 5C) after irradiation. Earlier findings implicated pericryptal COX2-positive cells in intestinal growth and adaptation (27, 28), though overall stromal COX2-positive cell abundance was comparable by genotype (Supplemental Figure 6B). These findings together suggest that *Rbm47-IKO* mice adapt to radiation injury through pathways reflecting upregulated RNAs encoding stem cell markers and proliferative and antioxidative genes and potentially by pericryptal COX2 signaling.

### Rbm47-IKO mice exhibit protection against dextran sodium sulfate–induced colitis.

**Figure 6:**
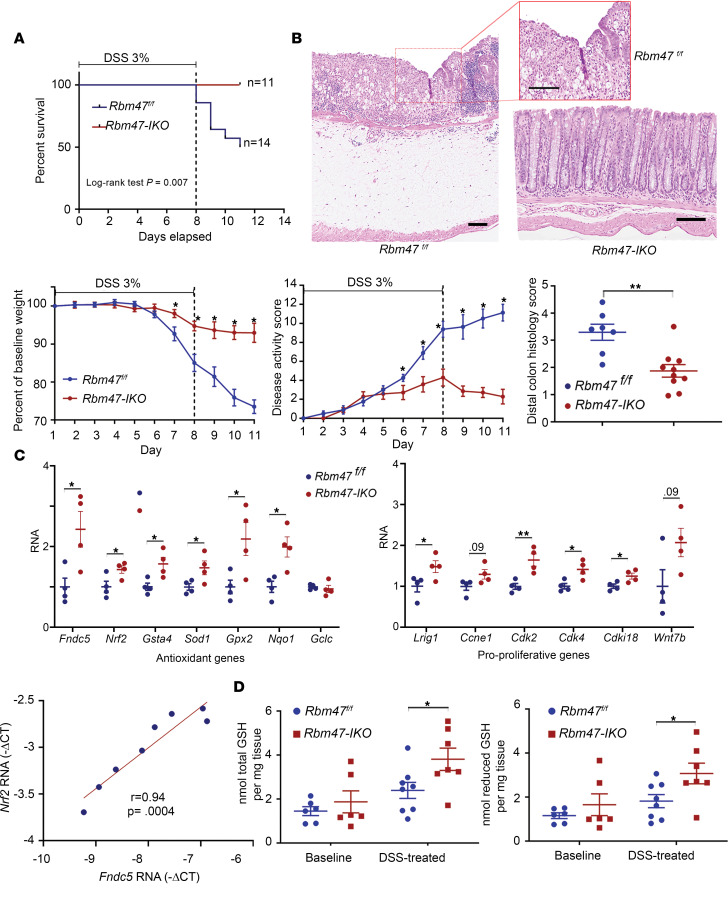
Constitutive intestinal *Rbm47* deletion attenuates colitis after 3% DSS treatment. (**A**) Survival rates (top), percentages of baseline body weight loss (bottom left), and disease activity scores (bottom right) of male *Rbm47^fl/fl^* and *Rbm47-IKO* mice treated with 3% DSS (unpaired *t* test, *n* = 14 *fl/fl* and 11 *IKO*); *P < 0.05. (**B**) Representative H&E-stained sections (top, scale bar: 100 μm) and microscopic histology score (bottom) of distal colon from *Rbm47^fl/fl^* and *Rbm47-IKO* mice treated with 3% DSS (mean ± SEM, unpaired *t* test, *n* = 7 *fl/fl* and 10 *IKO*); **P < 0.01. (**C**) qPCR evaluation of genes involved in antioxidative response and glutathione metabolism (top left), stem cell markers and cell proliferation genes (top right), and association between *Fndc5* and *Nrf2* expressions (bottom) in colons from DSS-treated male *Rbm47^fl/fl^* and *Rbm47-IKO* mice (mean ± SEM and unpaired *t* test for gene expressions, Pearson’s *r* test for association, *n* = 5/genotype); *P < 0.05, **P < 0.01. (**D**) Tissue content of total (left) and reduced (right) glutathione in distal colons from baseline and DSS-treated male *Rbm47^fl/fl^* and *Rbm47-IKO* mice (mean ± SEM, unpaired *t* test, *n* = 6/genotype for baseline and *n* = 8 *fl/fl* and 7 *IKO* for DSS group); *P < 0.05.

We then turned to an alternative model of intestinal injury, namely dextran sodium sulfate–induced (DSS-induced) colitis. *Rbm47-IKO* mice demonstrated significantly higher survival associated with attenuated body weight loss, lower disease activity score (Figure 6A), and consistently lower histologic injury score (Figure 6B) compared with floxed controls. We found upregulation of antioxidant genes, stem cell markers, and proliferative genes in colonic mucosa from *Rbm47-IKO* mice (Figure 6C). We also observed a significant correlation between *Fndc5* and *Nrf2* mRNA expression (*r* = 0.94, Figure 6C), similar to our observation in irradiated mice. In line with the upregulation of antioxidant genes, we observed increased colonic total and reduced glutathione content after DSS injury in *Rbm47-IKO* mice (Figure 6D). These findings suggest that *Rbm47-IKO* mice exhibit both reduced injury and more rapid recovery after DSS-induced colitis associated with upregulated antioxidative response and increased proliferative capacity.

### Rbm47-IKO mice exhibit spontaneous polyposis and increased polyp burden in compound Rbm47-IKO Apc^Min/+^ background.

**Figure 7:**
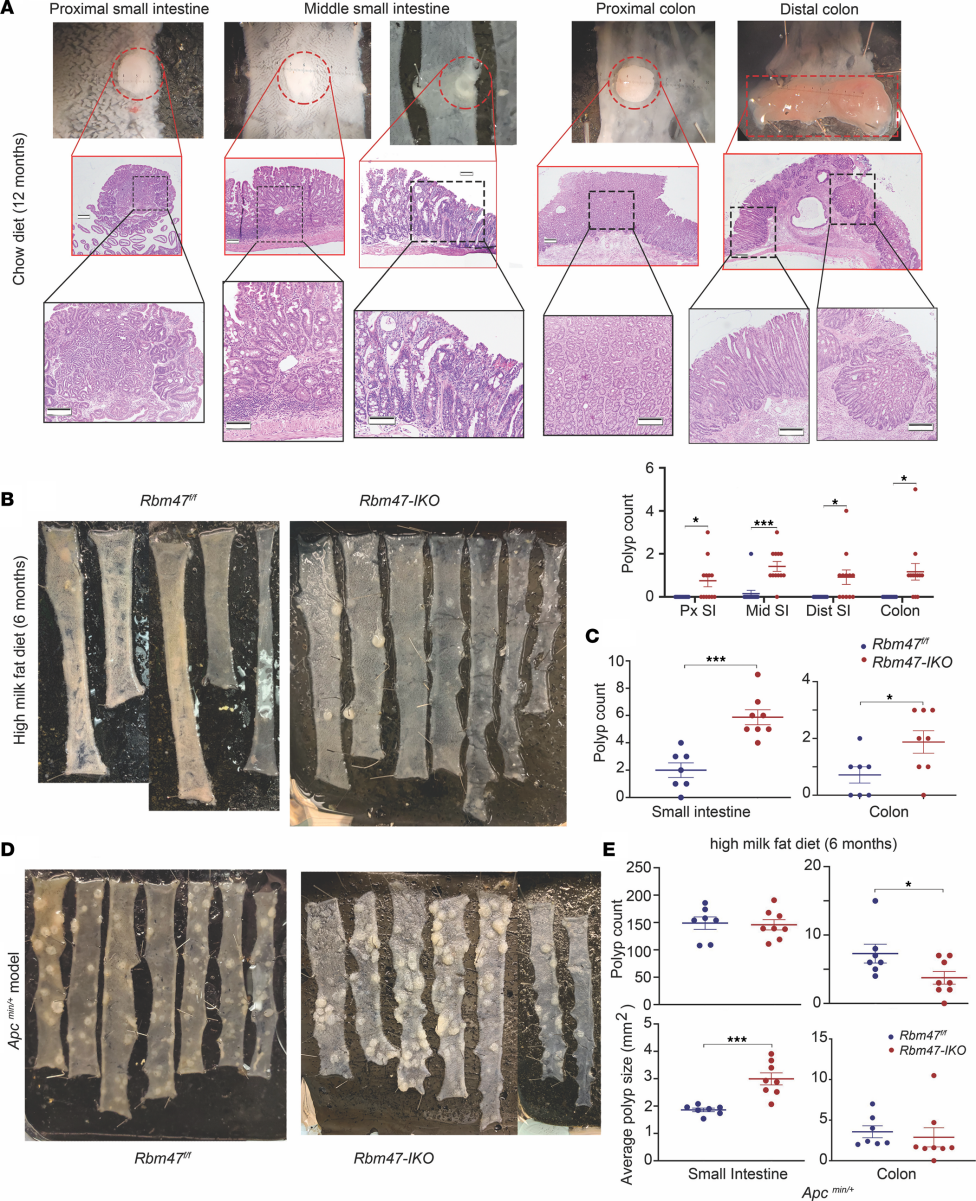
Constitutive intestinal *Rbm47* deletion promotes spontaneous and accelerated intestinal polyposis and results in bigger intestinal polyps in *Apc^Min/+^* mice. (**A**) Representative gross pictures (top) and H&E-stained sections with increasing magnifications (middle) of polyps in different segments of small intestine and colon from aged *Rbm47-IKO* mice on chow diet (scale bar: 100 μm) and regional distribution of polyps (bottom) in small intestine and colon from aged *Rbm47^fl/fl^* and *Rbm47-IKO* mice (mean ± SEM, unpaired *t* test, *n* = 13 *fl/fl* and 12 *IKO*); *P < 0.05, ***P < 0.001. (**B** and **C**) Representative gross pictures (**B**) and polyp count in small intestine (**C** left) and colon (**C** right) from *Rbm47^fl/fl^* and *Rbm47-IKO* mice fed with high–milk fat diet for 6 months (mean ± SEM, unpaired *t* test, *n* = 7 *fl/fl* and 8 *IKO*); *P < 0.05, ***P < 0.001. (**D** and **E**) Representative gross pictures (**D**), polyp count (**E** top), and average polyp size (**E** bottom) in small intestine (**E** left) and colon (**E** right) from *Apc^Min/+^* *Rbm47^fl/fl^* and *Apc^Min/+^ Rbm47-IKO* mice (mean ± SEM, unpaired *t* test, *n* = 7 *fl/fl* and 8 *IKO*); *P < 0.05, ***P < 0.001.

We reasoned that the enhanced intestinal growth and proliferative capacity in young *Rbm47-IKO* mice might predispose these animals to develop polyps with aging. To test this hypothesis, we examined aged (~12-month-old) chow-fed mice and observed increased small intestinal and colonic polyposis in *Rbm47-IKO* mice. The majority of these polyps were adenomatous, though 1 jejunal polyp demonstrated dysplastic features (Figure 7A). In all, 11 of 12 aged *Rbm47-IKO* mice developed polyps throughout the small intestine and colon, the dominant site being proximal and midjejunum, while only 1 out of 13 aged *Rbm47^fl/fl^* mice developed 2 polyps in the mid–small intestine (Figure 7A and Supplemental Figure 7A). We observed sporadic polyposis in different regions of *Rbm47-IKO* colon (Figure 7A). High-fat–fed *Rbm47-IKO* mice also exhibited enhanced polyposis (Figure 7B), with increased abundance of both small intestinal and colonic polyps (Figure 7C), with a total small intestine and colon polyp burden almost 3 and 2 times higher than controls, respectively. These observations suggest that the enhanced proliferative capacity observed in *Rbm47-IKO* mice promotes spontaneous polyposis with aging and is exacerbated by high-fat feeding.

Based on those observations, we asked if epithelial *Rbm47* deletion would function as a dominant driver of intestinal polyposis in the setting of adenomatous polyposis coli (*Apc*) loss, a hypothesis we examined by generating a line of *Rbm47-IKO* mice in the *Apc^Min/+^* background. We observed similar total numbers of polyps in both genotypes but found larger polyps and increased total polyp area in the small intestine of compound *Rbm47-IKO* *Apc^Min/+^* mice versus *Rbm47^fl/fl^* *Apc^Min/+^* mice (Figure 7, D and E), suggesting enhanced polyp progression in *Rbm47-IKO* *Apc^Min/+^* mice. Those small intestinal polyps in compound *Rbm47-IKO* *Apc^Min/+^* mice also exhibited dysplastic features (Supplemental Figure 7B). By contrast, colonic polyp count was reduced in *Rbm47-IKO* *Apc^Min/+^* mice, with no change in polyp size (Figure 7E). Those findings suggest that intestinal *Rbm47* deletion exerts regional effects on tumorigenesis in *Apc^Min/+^* mice. Immunohistochemical analysis of tumors from *Rbm47-IKO* *Apc^Min/+^* mice revealed patchy clusters of RBM47 staining, likely representing clonal expansion of cells no longer expressing the *Vil-Cre* transgene, while *Rbm47^fl/fl^* *Apc^Min/+^* mice revealed homogeneous RBM47 staining (Supplemental Figure 7C).

### Rbm47-IKO mice exhibit decreased colon tumorigenesis after azoxymethane/DSS.

**Figure 8:**
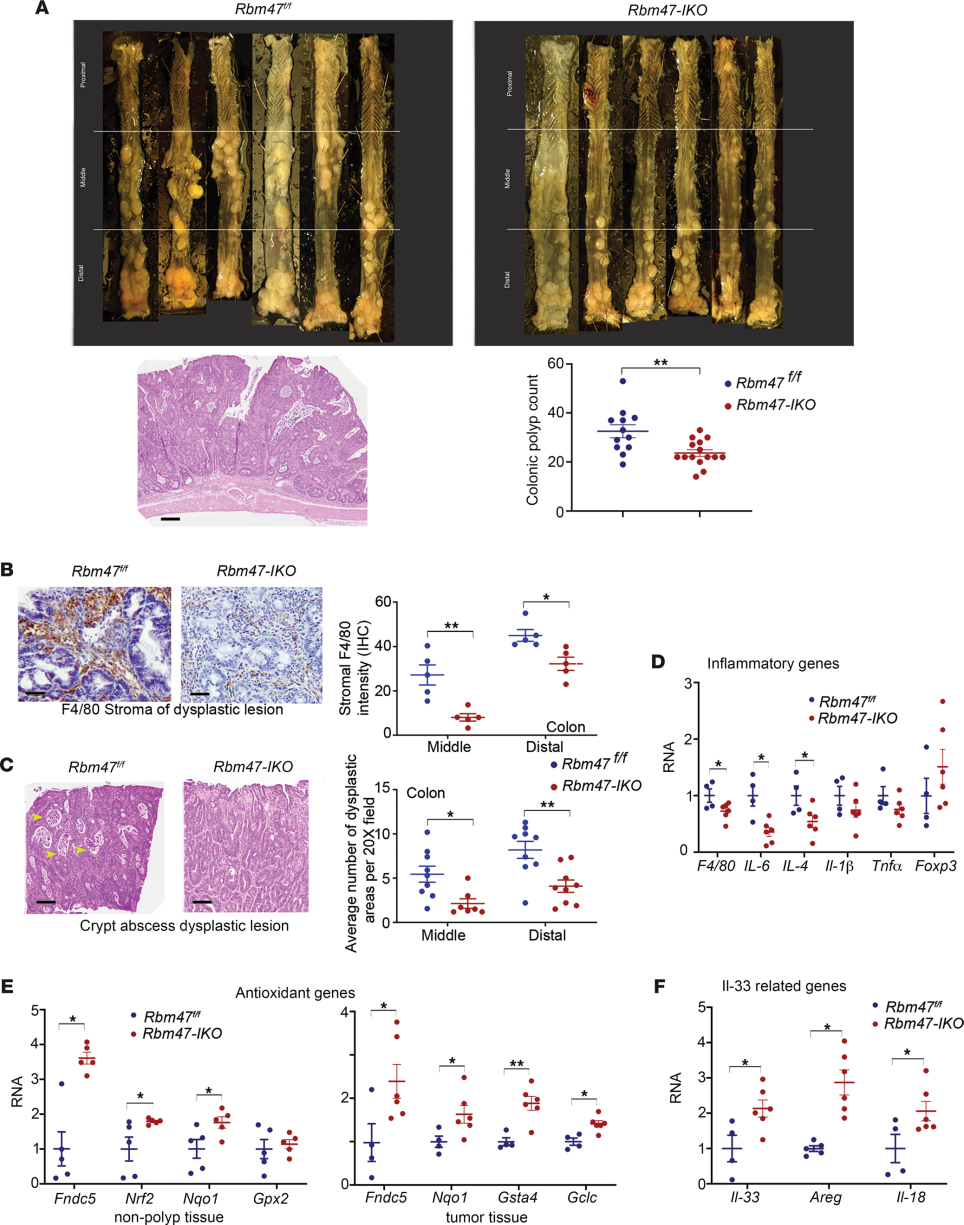
Constitutive intestinal *Rbm47* deletion mitigates colitis-associated tumorigenesis after AOM/DSS treatment. (**A**) Top: Representative gross images of colon from *Rbm47^fl/fl^* and *Rbm47-IKO* mice after AOM/DSS treatment. Bottom: H&E-stained section of a colonic polyp (left, scale bar: 250 μm) from a *Rbm47^fl/fl^* mouse and polyp count (right) from *Rbm47^fl/fl^* and *Rbm47-IKO* mice after AOM-DSS treatment (mean ± SEM, unpaired *t* test, *n* = 12 *fl/fl* and 15 *IKO*); **P < 0.01. (**B**) Immunohistochemical staining intensity for stromal F4/80 (left) and representative F4/80-stained sections (right, scale bar: 25 μm) of dysplastic colonic lesions from AOM/DSS-treated *Rbm47^fl/fl^* and *Rbm47-IKO* mice (mean ± SEM, unpaired *t* test, *n* = 5/genotype, 20 fields at original magnification 40× per mouse); *P < 0.05, **P < 0.01. Staining intensity was determined as the ratio of DAPI to hematoxylin optical density. (**C**) Average abundance of crypt abscess in dysplastic colonic lesions (left) and representative H&E-stained images (right, scale bar: 100 μm) of crypt abscess (yellow arrowhead) within dysplastic colonic lesions from AOM/DSS-treated *Rbm47^fl/fl^* and *Rbm47-IKO* mice (mean ± SEM, unpaired *t* test, *n* = 9 *fl/fl* and 7 *IKO*, 20 fields at original magnification 20× per mouse); *P < 0.05, **P < 0.01. (**D** and **F**) qPCR evaluation of inflammatory genes (**D**) and Il-33–related genes (**F**) in dysplastic colonic tissues from AOM/DSS-treated *Rbm47^fl/fl^* and *Rbm47-IKO* mice (mean ± SEM, unpaired *t* test, *n* = 5 *fl/fl* and 6 *IKO*); *P < 0.05. (**E**) qPCR evaluation of antioxidant genes in nondysplastic (left) and dysplastic (right) colonic tissues from AOM/DSS-treated *Rbm47^fl/fl^* and *Rbm47-IKO* mice (mean ± SEM, unpaired *t* test, *n* = 5 *fl/fl* and 6 *IKO*); *P < 0.05, **P < 0.01.

The findings that *Rbm47-IKO* mice exhibit enhanced small intestinal proliferative capacity and spontaneous polyposis, coupled with findings from patients with colorectal cancer (CRC) showing an association between loss of RBM47 and tumor progression (29), led us to ask whether intestinal *Rbm47* deletion might magnify the colonic tumor burden in a model of colitis-associated cancer. Alternatively, we considered the enhanced recovery and augmented antioxidative response with DSS injury in *Rbm47-IKO* mice might mitigate colitis-associated cancer and thus reduce the tumor burden. Our findings verified the latter suspicion. Indeed, we observed that *Rbm47-IKO* mice developed *fewer* tumors after challenge with azoxymethane (AOM)/DSS (Figure 8A). Histologic examination also showed reduced dysplasia in *Rbm47-IKO* mice, with 9/10 (90%) *Rbm47^fl/fl^* mice exhibiting dysplastic features in midcolon, versus 6/12 (50%) *Rbm47-IKO* mice (χ^2^ test *P* = 0.04) (Supplemental Figure 8A). Similarly, 6/10 *Rbm47^fl/fl^* and only 2/12 *Rbm47-IKO* mice exhibited dysplasia in more than 50% of distal colon length (χ^2^ test *P* = 0.03). Furthermore, dysplastic lesions from *Rbm47^fl/fl^* mice exhibited increased abundance of BrdU-positive cells (Supplemental Figure 8B). We next examined the stepwise processes that precede AOM/DSS-mediated tumorigenesis. We found that *Rbm47-IKO* mice demonstrated attenuated stromal immunohistochemical staining for F4/80 (Figure 8B) and reduced crypt abscesses, a surrogate histologic marker of inflammation (Figure 8C) in dysplastic colonic lesions. Likewise, *Rbm47-IKO* mice exhibited reduced inflammatory gene expression in polyp tissue (Figure 8D). To examine the mechanisms underlying attenuated inflammation in *Rbm47-IKO* mice, we interrogated relevant pathways directed by our RNA-Seq data and observed higher antioxidative mRNA expression in both uninvolved and polyp tissue in *Rbm47-IKO* mice following AOM/DSS administration (Figure 8E). Furthermore, we observed *Rbm47-IKO* mice exhibited upregulated mRNA expression of *Il-33* and its downstream targets, *Areg* and *Il-18*, both in baseline normal tissue and in colonic tumors after AOM/DSS (compare Figure 3H and Figure 8F). This observation is in line with earlier work showing a protective role of IL-33 and its downstream targets against chronic DSS-induced colitis (30, 31). Taken together, our findings suggest that the enhanced intestinal proliferative and protumorigenic phenotypes observed in *Rbm47-IKO* mice are counterbalanced by augmented antioxidative adaptations and upregulation of pathways including *Il-33* and its downstream targets that together protect these mice against inflammation and colitis-associated cancer after AOM/DSS treatment.

### RBM47 expression and alternative splicing function are downregulated with aging and in human patients with CRC.

**Figure 9:**
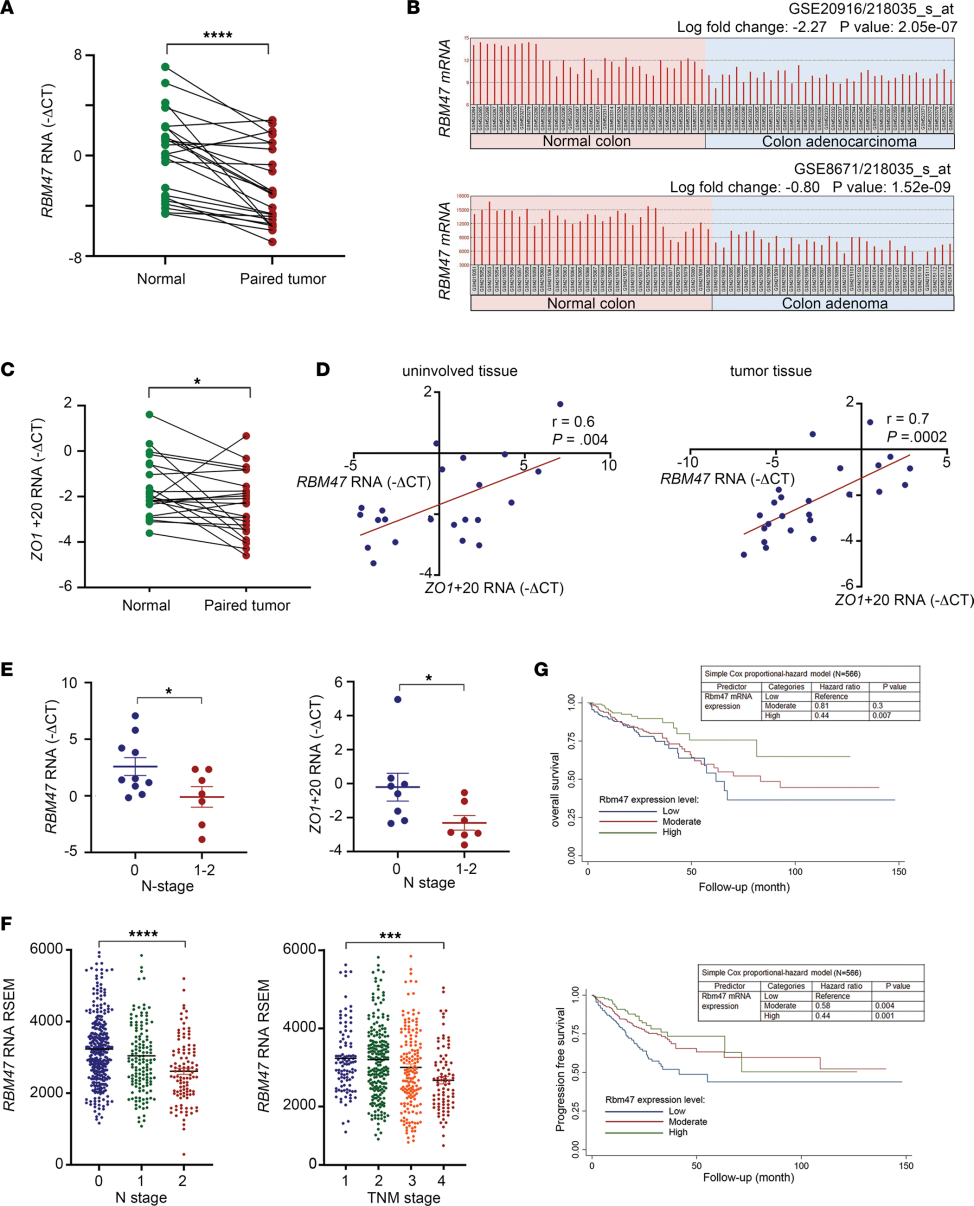
*RBM47* expression and alternative splicing of *Tjp1*/*Zo1* mRNA are downregulated with prognostic impact in patients with colorectal cancer. (**A**) *RBM47* mRNA expression in uninvolved and paired tumor tissue samples from patients with colorectal cancer (paired *t* test, *n* = 24); ****P < 0.0001. (**B**) *RBM47* mRNA expression in normal versus adenocarcinoma colon (top) (*n* = 34 normal and 36 adenocarcinoma) and normal versus adenoma colon (bottom) (*n* = 32 normal and 32 adenoma) samples extracted from NCBI GEO repositories GSE20916 and GSE8671, respectively. (**C**) qPCR evaluation of *Zo1+20* expression in uninvolved and paired tumor tissue samples from patients with colorectal cancer (paired *t* test, *n* = 22); *P < 0.05. (**D**) Correlation of *Rbm47* and *Zo1+20* expressions in uninvolved (left) and tumor (right) tissue samples from patients with colorectal cancer (Pearson’s *r* correlation test, *n* = 22 uninvolved and 24 tumor tissue samples). (**E**) *RBM47* (left) and *Zo1+20* (right) mRNA expressions in colorectal cancer patients with low, 0, and high, 1–2, N stage (mean ± SEM, unpaired *t* test, *n* = 8–10 low stage and 7 high stage); *P < 0.05. (**F**) *RBM47* mRNA expression in colorectal cancer patients with different N (left) and TNM (right) stages (mean ± SEM, ANOVA test, N stage *n* = N0 340, N1 141, N2 108; TNM stage *n* = TNM1 103, TNM2 220, TNM3 170, TNM4 85); ***P < 0.001, ****P < 0.0001. RSEM, RNA-Seq by Expectation Maximization software. (**G**) Overall (top) and progression-free (bottom) survival rates of colorectal cancer patients with different levels of tumoral *Rbm47* mRNA expression (Kaplan-Meier curves and simple Cox proportional-hazard model, *n* = 141 low expression, 282 moderate expression, 141 high expression). For **F** and **G**, data were extracted from PanCancer colorectal adenocarcinoma database of The Cancer Genome Atlas.

We next explored the role of RBM47 expression and function in deidentified human patients with CRC and found that *RBM47* mRNA was significantly downregulated in cancer tissue compared with paired uninvolved tissue (Figure 9A). In silico data sets from the National Center for Biotechnology Information (NCBI) Gene Expression Omnibus (GEO) further revealed that RBM47 was downregulated in both human adenocarcinoma and adenoma tissue compared with paired uninvolved tissue (Figure 9B). Prior work showed that RBM47 functions in alternative splicing of the mRNA encoding tight junction protein 1 (Tjp1/Zo1), with alternative transcripts either including (Tjp1+E20) or skipping (Tjp1–E20) exon 20 (Supplemental Figure 9A), consistent with the proposal that alternative splicing may promote epithelial-mesenchymal transition, as described in other settings (32). Similarly, we found reduced abundance of Tjp1+E20 mRNA in both intestinal mucosa and enteroids from *Rbm47-IKO* mice and observed a further decrease in spontaneous polyp tissue versus uninvolved mucosa in aged *Rbm47-IKO* mice (Supplemental Figure 9B), indicating altered splicing in tumors. Among the possible targets of the TJP1 isoform lacking exon 20 is ZONAB, a Y-box transcription factor whose nuclear translocation results in upregulation of pro-proliferative genes *Cdk4*, *Erbb2*, and *Ccnd1* (33–35), all of which were upregulated in *Rbm47-IKO* mice as shown above (Figure 4, H and J), consistent with a proposed model linking alterations in tight junction–associated proteins and intestinal growth (35). Similar to our findings in aged mice, we found decreased abundance of TJP1+E20 in paired CRC tumors versus normal tissue, indicating alternative splicing in human tumors (Figure 9C). We also demonstrated that TJP1+E20 mRNA expression was significantly and positively associated with *Rbm47* expression in both uninvolved and CRC tissue from patients (Figure 9D). Furthermore, we observed decreased *RBM47* and TJP1+E20 mRNA expression in tumors from patients with N stage 1–2 versus N stage 0 (Figure 9E). These findings together suggest that decreased RBM4*7* in human CRC is associated with progressive changes in alternative splicing of *TJP1* during CRC progression. Further analysis of The Cancer Genome Atlas (TCGA) database revealed a stepwise reduction of RBM47 expression with advancing tumor stage (Figure 9F), which was independently predictive of both overall and progression-free survival (Figure 9G). A prognostic role of RBM47 expression in patients with CRC remained significant even after adjusting for other important prognostic factors, such as TNM stage, age, and sex, through a multivariable Cox survival model (Table 1).

## Discussion

The current findings establish a role for the RBP RBM47 in intestinal epithelial homeostasis, including cell type differentiation, proliferation, adaptation to injury, and tumorigenesis. We show that *Rbm47-IKO* mice exhibit a distinctive phenotype of small intestinal elongation, increased growth, and altered villus morphology, with a shift in cellular composition. In addition, we find that *Rbm47-IKO* mice exhibit adaptive responses to both radiation-induced injury and experimental colitis induced by DSS exposure, along with reduced colitis-associated tumorigenesis. In line with the increased growth and proliferation phenotypes, we find that *Rbm47-IKO* mice exhibit spontaneous polyposis with aging and an exaggerated polyposis phenotype in the *Apc^Min/+^* background. Those findings were further amplified by the demonstration that RBM47 expression is reduced in human CRC samples and exerts a positive prognostic effect. RNA-Seq and mechanistic assays suggested increased cell proliferation in *Rbm47-IKO* mice reflects altered WNT signaling, while upregulation of FNDC5/NRF2-driven antioxidative activity is a plausible mechanism underlying the adaptation to inflammatory injury observed following DSS exposure. Taken together, our findings highlight a role for RBM47 as a cell-intrinsic modifier of small intestinal and colonic epithelial homeostasis and illuminate new pathways in the initiation of tumorigenesis. Several aspects of these findings merit expanded discussion.

Earlier work demonstrated that RBM47 plays a vital role in embryonic foregut, including epithelial organization and differentiation, effects mediated in part through altered F-actin distribution (3), while later studies demonstrated a critical role for RBM47 in embryonic and postnatal development (5, 6). The gut phenotypes associated with embryonic *Rbm47* disruption using Sox2-Cre, in particular the prenatal lethality and runting in small numbers of surviving pups (5, 6), contrast with the current findings following intestinal epithelial deletion using Villin-Cre (Vil-Cre) recombinase, which is active in the midgut at E12.5 (36). The morphologic and proliferative phenotypes we observed in young adult *Rbm47-IKO* mice thus likely reflect both the later onset (E12.5 with Vil-Cre versus E6.5 with Sox2-Cre) and cell-specific Cre-mediated *Rbm47* deletion approaches compared with conditional *Rbm47* deletion with Sox2-Cre (5, 36, 37). Similar contrasting phenotypes were observed with conditional deletion of *HuR* where intestinal epithelial (i.e., Vil-Cre) deletion yielded a subtle growth phenotype while the global *HuR* knockout (Rosa-Cre) demonstrated progressive villus atrophy and death (38, 39). Our observations that enteroids and colonoids from *Rbm47-IKO* mice recapitulated the proliferative phenotypes demonstrated in vivo lend further support for a cell-intrinsic role for epithelial RBM47.

We identified a range of mRNAs whose expression was altered in the setting of intestinal *Rbm47* deletion, including genes involved in *Wnt* signaling (including *Wnt11*, *Fzd8*, *Fzd23*, *Ccdn1*, *Cdk2*), as well as stem cell and developmentally regulated genes (*Lrig1*, *Epcam*, *S-100*, *Hoxa3*, *Hoxb13*, *Epop*, and *A2ml1*). Those findings reinforce suggestions that RBM47 is both a downstream target of *Wnt* signaling as well as a feed-forward activator of *Wnt* pathways (13). Enteroids derived from *Rbm47-IKO* mice grow in a cystic pattern reminiscent of a fingerprint of augmented WNT signaling (18, 19), supporting a role for this pathway in the enhanced proliferative capacity. In addition, upregulation of stem cell markers (*Lrig1* and *Lgr5*) in *Rbm47-IKO* mice, either at baseline or after injury, suggests that enhanced activity of intestinal stem cells further contributes to the pro-proliferative and tumorigenic phenotypes observed. Intestinal scRNA-Seq data (17) revealed relatively reduced *Rbm47* mRNA abundance in stem cells and transitional amplifying cells compared with differentiated enterocytes, consistent with the proposed association between *Rbm47* expression and differentiation of murine intestinal cells.

Other significant mechanistic signatures underlying phenotypes observed in *Rbm47-IKO* mice include FNDC5/NRF2-driven augmentation of antioxidative pathways. *Fndc5* is one of the top upregulated genes from RNA-Seq in *Rbm47-IKO* mice and associated with higher abundance of FNDC5 protein. *Rbm47-IKO* mice exhibit increased stability of *Fndc5* mRNA and shifts in polysomal distribution, promoting increased mRNA abundance and augmented translation/protein expression. Since NRF2 is a key upstream regulator of antioxidative pathways, we conclude that upregulation of *Fndc5* mRNA and protein in *Rbm47-IKO* mice results in upregulation of many downstream antioxidative genes through *Nrf2* upregulation. Augmented antioxidative gene expression and activity observed in *Rbm47-IKO* mice were further validated by increased tissue content of glutathione. Taken together, these data demonstrate augmented antioxidative pathways in *Rbm47-IKO* mice, driven by upstream regulator genes, *Fndc5* and *Nrf2*. However, it will be important to define in more detail the downstream signaling pathways involved.

Our findings highlight consensus features in the 3′ untranslated regions of the most highly regulated transcripts, which are broadly consistent with other studies using CLIP-Seq (2, 16). Further study will be required to substantiate a specific set of target RNAs for intestinal RBM47, and we acknowledge that our analysis, based on mucosal extracts, which contain a mixture of epithelial, stromal, muscle, and vascular endothelial cells, may not offer sufficient sensitivity to resolve enterocyte-specific pathways. For example, RBM47 was shown to modulate expression of IL-10 in B lymphocytes, and overexpression of RBM47 in B lymphocytes conferred protection against colonic epithelial injury in mice following DSS administration (20). Those findings, along with the current findings, suggest that both gain- and loss-of-function phenotypes may be associated with altered *Rbm47* expression, depending on the cell context. In addition, it is likely that RBM47 interacts in multiple combinatorial models involving protein-RNA and protein-protein-RNA complexes (7) whose composition reflects both direct and indirect interactions.

RBM47 has also been implicated in aspects of somatic cell reprogramming, specifically as a regulator of alternative RNA splicing (40). Other work showed upregulation of microRNAs (miR186, 199a, 339), with terminal differentiation of pancreatic islet cells associated with loss of RBM47 expression (41), suggesting that regulated loss of RBM47 expression may function in a developmentally regulated signaling cascade during foregut development. Loss of RBM47 expression is also associated with epithelial-mesenchymal transition, via alternative splicing of *TJP1* mRNA (32). We speculate that alternative splicing of TJP1 alters proliferative activity through pathways including regulating nuclear translocation of ZONAB, a Y-box transcription factor that targets multiple pro-proliferative genes, such as *Cdk4* and *Ccnd1* (33–35).

A further aspect of our findings raises the question of how certain loss-of-function phenotypes following intestinal *Rbm47* deletion can be internally reconciled. More specifically, we observed seemingly disparate findings with protection against injury and colitis-associated cancer despite increased spontaneous tumorigenesis. Possible components of this dilemma reside in the different roles and importance of inflammation- versus oxidation-associated injury as driver events as well as the role of stromal and other sources of signaling molecules. As discussed above, *Rbm47-IKO* mice exhibit augmented antioxidative capacity, which confers protection from inflammation-associated and oxidative injury in colitis-associated carcinogenesis. However, attenuated inflammatory and oxidative injury may be less relevant than upregulation of cell proliferation, particularly in the setting of spontaneous polyposis in *Rbm47-IKO* mice.

Another explanation for the different tumorigenesis pattern in *Rbm47-IKO* mice may reside in the contradictory roles of IL-33/AREG axis signaling in different models. We observed upregulation of the IL-33/AREG axis in *Rbm47-IKO* mice, a pathway previously shown to promote polyposis in *Apc^Min/+^* mice (42, 43). On the other hand, studies have demonstrated that IL-33 signaling via AREG represents a dominant pathway for gut-associated group 2 innate lymphoid cells, whose activation promotes *protection* against colitis and colitis-associated cancer (43). Those latter findings are consistent with yet other findings showing that *Il33*-null mice are more susceptible to DSS-induced injury and colitis-associated cancer (30). Taken together with the current findings showing increased IL-33 and AREG expression in *Rbm47-IKO* mice, we propose that these adaptations might plausibly protect against intestinal injury and inflammation-associated tumorigenesis while promoting spontaneous polyposis. Along these lines, recent work has demonstrated the importance of tumor cell differentiation status in shaping the adaptive immune environment in the *Apc^Min/+^* model, as well as in human colonic adenomas (44). Further study will be necessary to understand the role of altered stromal and microbial signaling in these distinct tumorigenic phenotypes in *Rbm47-IKO* mice.

Other plausible mechanisms linking RBM47 and carcinogenesis include IL-6/STAT3 signaling, which has been associated with decreased RBM47 expression (29). Those findings suggest that loss of RBM47 may arise as a *consequence* of inflammation during cancer progression in addition to cell-intrinsic pathways of downregulation associated with tumor initiation. Downregulation of RBM47 is observed with altered promoter methylation in neuroendocrine pancreatic tumors (45), suggesting yet another mechanism underlying a loss-of-function role in tumorigenesis. With the increasing burden of obesity and its pleiotropic effects on inflammation and cancer (46), it may become increasingly challenging to parse out distinct pathways in a physiological, in vivo setting.

In summary, our findings implicate RBP RBM47 as a cell-intrinsic modifier of intestinal growth, inflammatory, and tumorigenic pathways. Further resolution of the range of cell-specific targets and their function may highlight signaling pathways relevant to tumorigenesis in the setting of both inflammatory conditions such as Crohn’s disease and ulcerative colitis, as well as sporadic CRC.

## Methods

### Animals and husbandry.

Mice were housed in ventilated cages on a 12-hour light/12-hour dark cycle with corncob bedding and ad libitum access to rodent chow (PicoLab Rodent Diet 20, LabDiet) and water unless otherwise noted. *Rbm47^fl/fl^* and *Rbm47-IKO* lines were generated on a C57BL/6J background, as described previously (7). The studies used constitutive Vil-Cre [B6.Cg-Tg(Vil1-cre)1000Gum/J], The Jackson Laboratory 021504. We also used the tamoxifen-inducible Vil-Cre [B6.Cg-Tg(Vil1-cre/ERT2)23Syr/J], The Jackson Laboratory 020282 (47), to generate inducible *Rbm47-IKO* mice. Because inducible and constitutive *Rbm47-IKO* mice exhibited similar phenotypes at baseline, further experiments were done only on the constitutive *Rbm47-IKO* line. Studies show that the Vil1Cre/1000 transgene is expressed extensively in small and large intestine, with only scattered expression in the urogenital tract (48). Studies were performed on chow-fed mice in the range of 8–14 weeks or where indicated in about 12-month-old mice. Where indicated, mice were fed a high–milk fat diet (TD.09766, Envigo Teklad) for 6 months prior to study. Mice received 100 mg/kg BrdU (MilliporeSigma) 2 hours before harvest. *Rbm47^fl/fl^* mice were used as controls in all experiments.

### Mouse irradiation and microcolony assay.

Mice received 12 Gy irradiation in a Gammacell 40 137Cs irradiator at 80.7 cGy/min. Mice were sacrificed at 84 hours, with crypt survival measured using a modified microcolony assay (21, 22). Each mouse was given a mixture of BrdU (120 mg/kg) and 5-fluoro-2-deoxyuridine (12 mg/kg) 90 minutes before sacrifice. BrdU was detected using rat anti-BrdU (1:200; catalog ab6326; Abcam), then developed with DAB (MilliporeSigma). Cross sections of proximal jejunum were used for the microcolony assay. The viability of each surviving crypt was confirmed by incorporation of BrdU into 6 or more epithelial cells within each regenerative crypt. Eight complete cross sections were scored for each mouse. The intestinal sections were also examined for abundance of pericryptal COX2–positive cells. To evaluate apoptosis, groups of mice were sacrificed 6 hours after irradiation. TUNEL-stained sections from jejunal crypts were used to compare apoptosis after irradiation between 2 genotypes.

### DSS colitis model.

Male 10- to 12-week-old mice were administered 3% DSS (TdB Labs) water for 7 days (day 1–8). On day 8, the mice were switched to tap water and followed for 3 more days before harvest on day 11. Body weight, rectal bleeding, and stool consistency were monitored before and during the treatment daily to calculate disease activity score (maximum = 12, Supplemental Table 1). Death or body weight loss more than 20% were considered terminal outcomes in the survival analysis. After harvest, H&E-stained distal colon was examined for determining histologic injury. The injury score (49, 50) was based on the extent of damage (mild, moderate, severe) in 3 compartments: epithelium architecture (hyperplasia/disarray, sloughing/crypt loss, ulcer), mucosal infiltration/inflammation (scattered, dense, lymphoid aggregates), and submucosal inflammation/infiltration (edema or infiltration).

### AOM/DSS treatment.

Mice (5–6 weeks old) were injected intraperitoneally with 10 mg/kg AOM on day 1 and day 3 and otherwise studied as described (38). One week after the second injection, mice were treated with 2.5% DSS water for 1 week, followed by 2 weeks of tap water. The cycle of 1 week DSS water and 2 weeks tap water was repeated 2 more times (total 3 cycles, 9 weeks). Mice were harvested at the end of the third cycle (day 73). After harvest, gross polyp count was examined, and H&E-stained colonic sections were surveyed for length of colon (%) involved with dysplasia graded by a pathologist under a blinded experimental protocol. The abundance of crypt abscess within the dysplastic lesions and stromal immunohistochemical staining for F4/80 were also evaluated.

### Generation of Rbm47^fl/fl^ Apc^Min/+^ and Rbm47-IKO Apc^Min/+^ mice.

Animals were generated by crossing heterozygote C57BL/6J *Apc^Min^*/J males (002020, The Jackson Laboratory) with either *Rbm47^fl/fl^* or *Rbm47-IKO* females. Genotyping for *Rbm47^fl/fl^* and *Rbm47-IKO* was performed as described previously (7). Genotyping for *Apc^Min/+^* was performed using the following primers: MAPCMT (P1) (5′-TTCTGAGAAAGACAGAAGTTA-3′), MAPC15 (P2) (5′-TTCCACTTTGGCATAAGG-3′), and MAPC9 (P3) (5′-GCCATCCCTTCACGTTAG-3′), using the following conditions: 94°C for 3 minutes; 94°C for 30 seconds, 60°C for 2 minutes, 72°C for 3 minutes (30 cycles); 72°C 10 minutes. The PCR generates a 618 bp wild-type band and a 327 bp Apc^Min^ band. All animals were maintained on a breeder diet (S-2335, Envigo Teklad), as described (51).

### Tissue content of glutathione.

Intestinal scraped mucosa and full-thickness colon tissue were used to evaluate tissue content of total and oxidized glutathione following manufacturer’s protocol (Cayman Chemical catalog 703002). Reduced glutathione was calculated by subtracting oxidized glutathione concentration from total glutathione concentration.

### Intestinal permeability.

Intestinal permeability was examined using FD4 (MW 4,000, MilliporeSigma). Control and *Rbm47-IKO* mice were gavaged with FD4 (400 mg/kg body weight), and sera were collected 4 hours later. Serum FITC was measured on a fluorometer (Synergy HT, BioTek) at excitation 485/20 nm, emission 528/20 nm (52).

### Electron microscopy.

Mice jejunum was cut along the mesenteric border, pinned, and rinsed thoroughly with Krebs-Ringer buffer. For scanning electron microscopy (SEM), jejunal samples were fixed using electron microscopy–grade (EM-grade) 2.5% glutaraldehyde and 2% paraformaldehyde (PFA) in 0.10 M cacodylate buffer with 2 mM CaCl_2_ at 37°C, then were allowed to postfix overnight at 4°C. Samples were subsequently rinsed in 0.10 M cacodylate buffer containing 2 mM CaCl_2_ (3 times, 10 minutes) and were processed as described (53) to reduce charging artifacts during imaging. Samples were then dried in a critical point dryer (Leica EM CPD300), mounted on SEM stubs, and coated with 48 nm iridium using a Leica EM ACE600 Sputter Coater. SEM samples were visualized on a Merlin Field Emission Scanning Electron microscope (Carl Zeiss) and acquired using an SE2 secondary electron detector at 2 kV, using a 200 pA probe. For transmission electron microscopy, jejunal samples were fixed using EM-grade 2.5% glutaraldehyde and 2% PFA in 0.10 M cacodylate buffer with 2 mM CaCl_2_ at 37°C for 1 hour, then stored in the fixative overnight at 4°C. Samples were subsequently rinsed in 0.10 M cacodylate buffer containing 2 mM CaCl_2_ (3 times, 10 minutes), postfixed with 1% OsO_4_/1.5% potassium ferrocyanide in 0.10 M cacodylate buffer containing 2 mM CaCl_2_ for 1 hour, washed in MilliQ water (MilliporeSigma; 3 times, 10 minutes), and en bloc–stained in aqueous 2% uranyl acetate (UA) 1 hour in dark. Samples were rinsed in MilliQ water (3 times, 10 minutes); dehydrated in an ethanol series consisting of 30%, 50%, 70%, 90%, 100% 3 times for 10 minutes each; infiltrated with resin through an Epon/ethanol series of 30%, 50%, 70%, 90%, 100% (3 times with microwave assistance); and cured at 60°C for 72 hours. Sections 70 nm thick were cut on a Leica EM UC7 ultramicrotome, counterstained in 2% aqueous UA and Sato’s lead (54), then imaged with a JEOL JEM-1400Plus transmission electron microscope. For 3D x-ray topographic imaging, samples were fixed overnight in 4% PFA at 4°C and were incubated in 1% I2KI Lugol’s Iodine for 5 days. Samples were embedded in a 2% agarose gel and were visualized on a Versa 520 x-ray microscope (Carl Zeiss). Data sets were acquired using a source tuned to 80 keV and either a flat panel (0.4×) or 20× objectives, and tomograms were generated using ORS Dragonfly 2020.2.

### Histology and immunohistochemistry.

For polyp quantification, small intestine and colon were gently dissected and tissues pinned. Two independent observers blinded to genotype quantified the polyps, with consensus achieved in the case of discrepancy. For embedding, mouse intestine and colon were pinned and fixed with neutral formalin overnight, followed by embedding in 2.5% agarose. The embedded sections were cut into 4 μm tissue layers mounted on glass slides for staining. For evaluation of colon mucus layer thickness, colon segments were immediately removed and fixed in ice-cold Carnoy’s solution (ethanol 6: acid acetic 3: chloroform 1, vol/vol) for 2 hours at 4°C. They were then immersed in ethanol 100% for 24 hours at 4°C. H&E-stained intestinal and colonic slides were evaluated with representative views of 30–40 villus and crypt units examined per mouse. Villi with distinctly abnormal shape (e.g., bifurcating or mushroom shaped) or with a width more than the 95th percentile were considered abnormal villi. Crypts in their early stage of duplication and bifurcation were considered as crypt fission. The incidences of abnormal villi and crypt fission were evaluated by examining 200 villus and crypt units per mouse, respectively. Pathologists unaware of the experimental groups performed the histologic survey of the intestinal and colonic polyps (adenomatous versus dysplastic) on the H&E-stained slides. Average abundance of BrdU-positive cells in the crypt was calculated by examining 20–30 crypts per mouse. The percentage of Alcian blue–positive cells was examined in 30–40 sections with complete longitudinal cross-sectional views of villi and crypts. Periodic acid–Schiff was used to stain colon mucus layer. A minimum of 20 measurements were made perpendicular to the inner mucus layer per field, and 10 randomly selected fields were analyzed for each colon. Immunohistochemical analysis was undertaken using anti–cyclin D1, anti-Dclk1 (for detection of tuft cells), anti–Chromogranin A (for detection of enteroendocrine cells), anti-lysozyme (for detection of Paneth cells), anti-COX2, anti-F4/80, and anti–β-catenin antibodies. Antibody sources are as follows: cyclin D1: ab134175 (Abcam), 1:300; DCLK1: ab391704 (Abcam), 1:50; lysozyme: ab2408 (Abcam), 1:200; F480: 70076S (Cell Signaling Technology), 1:600; BrdU: ab152095 (Abcam), 1:500; and β-catenin: 610154 (BD Biosciences), 1:2,000. ImageJ software (NIH) was used to perform the calculations on the images taken with a light microscope from the stained slides. In the case of F4/80, immunohistochemical staining intensity was determined as the ratio of DAP optical density to hematoxylin optical density. Optical densities were calculated as the log (255/mean density measured by ImageJ).

### Organoid experiments.

Enteroids and colonoids were prepared from mouse jejunum and colon crypt stem cells, as described (19). For maintenance passage, organoids were dissociated using trypsin, washed with washing medium, and resuspended in Matrigel for plating. For colony-forming assay, organoids were dissociated more vigorously to single or diploid cells. Calculated volume of the final cell suspension was used to reach 5,000 enteroids and 15,000 colonoids per well. The number of organoids, per well, 7 days postplating was counted using the Cytation 5 scanner (BioTek Instruments) and divided by the initial number of cells plated to calculate colony-forming efficiency (%). Proliferation in organoid cultures was examined using 10 μM EdU added to culture medium for 2 hours, after which samples were fixed in neutral formalin, suspended in prewarmed 2.5% agar, and transformed to molds, which were sectioned onto microscope slides. EdU-positive cells were visualized using the Click-iT Plus EdU Alexa Fluor 488 Imaging Kit (Thermo Fisher Scientific catalog C10632) and following the manufacturer’s protocol. Hoechst (1:2,000) was used for nuclear counterstaining, and ProLong Diamond Antifade Mountant (Thermo Fisher Scientific catalog P36961) was used to prolog the fluorescence signal from the stained slides. Axiovert 200 microscope and Axiocam MRM camera with an Apotome optical sectioning filter (Carl Zeiss) were used to acquire images. ImageJ was used to quantify EdU-positive cells and total nuclei on the acquired images. To evaluate organoid cell viability, cell counting kit-8 (Dojindo Molecular Technologies, Inc. catalog SKU CK04) was used per the manufacturer protocol. Two days after plating, kit solution was added to the organoid culture medium (1:11) followed by 4 hours of incubation at 37°C. The absorbance at 450 nm was measured using a microplate reader. To examine *Fndc5* mRNA stability, enteroids and colonoids were exposed to 1.75 μg/mL and 5 μg/mL of actinomycin D (Thermo Fisher Scientific 11805017), respectively (experimentally determined as optimal doses) in the conditioned media, as described (55). Organoids were collected at baseline and at the indicated time points. *Fndc5* mRNA was quantified by qPCR at different time points and expressed as the proportion of the baseline *Fndc5* mRNA level.

### RNA extraction, cDNA synthesis, PCR, and qPCR.

TRIzol (Invitrogen catalog 15596026) was used per manufacturer’s instructions. Lithium chloride was added to the RNA solution in DSS-treated samples to a final concentration of 2.5 M, and the samples were chilled at –20°C for 30 minutes followed by centrifugation at 16,000*g* for 15 minutes at 4°C. The supernatant was discarded and the pellet washed with ice-cold ethanol (70%) followed by resuspension in nuclease-free water. RNA was extracted from organoids following removal of the Matrigel dome and transferred to 15 mL tubes on ice. The cell pellet was resuspended in 1 mL ice-cold TRIzol and transferred to a –80°C freezer. Total RNA was treated with DNase and then used to prepare cDNA with a High Capacity cDNA Reverse Transcription kit (Applied Biosystems). PCR products were generated from cDNA using Taq DNA polymerase (Invitrogen), and UV-illuminated PCR bands were captured for analysis. qPCR was performed in triplicate using Fast SYBR Green Master Mix (Applied Biosystems) and in a StepOne Plus Real Time PCR machine (Applied Biosystem). Total mRNA levels were expressed either as fold change or –ΔCT by normalization to *Gapdh* RNA in mouse and organoid samples and *Gusb* RNA in human samples. Supplemental Data File 1 lists the sequences for primers used for qPCR.

### RNA-Seq.

For each genotype 3 pools were prepared, each containing a total of 10 μg RNA from 3–4 separate mice. The pools were subjected to oligo deoxy-T selection, cDNA library preparation, and transcriptome sequencing by Illumina HiSeq 2000. Processing of the sequencing data, alignment of the RNA-Seq reads, and expression estimation of known Ensembl transcripts were performed as previously described (56). Benjamini-Hochberg false discovery rate–adjusted *P* values ≤ 0.05 were considered to filter significant differential expression of genes between genotypes. Genes with highly differentiated expressions were outlined by a heatmap generated using Java Treeview (57). To identify functional pathway enrichment within differentially expressed genes, the Database for Annotation, Visualization, and Integrated Discovery tool (58, 59) and STRING network analysis (https://string-db.org/) were used.

### Protein extraction and Western blotting.

Total protein was extracted from scraped intestinal mucosa as described (38). Wells with confluent organoids were washed with PBS. Matrigel-embedded organoids were scratched out of the wells into Cell Recovery Solution (Corning) and kept on ice for 45 minutes. Eluted organoids were washed in PBS, resuspended in cell lysis buffer (20 mM Tris pH 8, 50 mM NaCl, 2 mM EDTA, 1% Triton X-100, 5% glycerol, 100 mM NaF, 1 mM Na vanadate, 50 mM β-glycerophosphate 1× protease inhibitors), and disrupted through an 18-gauge needle. Total protein extract was recovered by centrifugation at 25,200*g*, at 4°C, for 10 minutes. Glass Dounce Tissue homogenizer was used to prepare the nuclear extract (7). The nuclear pellet was resuspended in 80 μL of buffer B (25 mM Tris pH 7.5, 420 mM NaCl, 1.5 mM MgCl_2_, 0.5 mM EDTA, 1 mM DTT, 20% glycerol, 1× protease inhibitor) and incubated in ice for 30 minutes. The suspension was centrifuged at 11,200*g* for 15 minutes at 4°C to collect the nuclear protein fraction. For Western blotting, 50 μg of total protein and 15 μg of nuclear protein were run on 10% and 12.5% SDS-PAGE gels, transferred to PVDF membranes, and probed with rabbit anti-RBM47 (Abcam ab94638; 1:2,000) and rabbit anti-FNDC5 (Abcam ab174833; 1:1,000) antibodies. Rabbit anti-actin (MilliporeSigma; ab179467; 1:2,000) antibody was used to confirm equal loading of protein.

### Data availability.

The RNA-Seq data used and discussed in this study are deposited in the NCBI GEO, accession number GSE206461. TCGA data set was downloaded from https://www.cbioportal.org/study/summary?id=coadread_tcga_pan_can_atlas_2018 Mouse intestinal scRNA-Seq data were derived from ref. 17 and analyzed using Single Cell Portal available at https://singlecell.broadinstitute.org The remaining data are available within the article or supplemental information (17).

### Statistics.

Data are shown as mean ± standard error of mean. *P* value less than 0.05 was considered statistically significant. One-way ANOVA and 2-tailed *t* test were used to compare the mean values across groups, and χ^2^ test was used to compare the distribution of categorical parameters across groups. We used Pearson’s correlation test to examine the association between continuous parameters. Mouse intestinal scRNA-Seq data were used to investigate *Rbm47* mRNA expression in different murine intestinal cells (17). *Rbm47* mRNA expression in human normal, adenoma, and adenocarcinoma colon tissue was extracted from NCBI’s GEO repositories GSE20916 (60) and GSE8671 (61). Furthermore, clinical and gene expression data for 566 patients with CRC were downloaded from TCGA PanCancer colorectal adenocarcinoma database (62–64) through the cBioPortal platform (65, 66). *Rbm47* mRNA expression was compared across CRC patients with different N and TNM stages. Kaplan-Meier curves and simple Cox proportional-hazard analysis were used to examine the association of *Rbm47* expression with overall and progression-free survival. We also used multivariable Cox modeling to adjust the prognostic significance of *Rbm47* expression in patients with CRC for other important prognostic factors, such as TNM stage. GraphPad Prism 9 (GraphPad Software Inc.) and StataMP 17 (StataCorp LP) were used for the statistical analyses.

### Study approval.

Access to the tissue samples from Digestive Diseases Research Core Center (DDRCC) for patients with CRC was approved by the Institutional Review Board of Washington University School of Medicine in St. Louis (IRB 201111078 Washington University DDRCC Biobank). Patients provided written informed consent for the tissue sampling. Deidentified uninvolved and cancer tissue samples from patients with CRC were obtained from the tumor biobank of the DDRCC at Washington University in St Louis. All patients provided informed consent for the collection and use of samples as approved by the Washington University in St. Louis Institutional Review Board as noted above. These samples were collected during the surgery, placed directly into RNALater, kept on the bench at room temperature overnight, and then stored in the –80°C freezer until use. All animal studies were approved by the Washington University in St. Louis Institutional Animal Care and Use Committee (IACUC 20190062 and 21-0276).

## Author contributions

SS and VB designed, conducted, and analyzed experiments. EAM, DMA, YX, and VG conducted and analyzed experiments. JWB, KB, and TCL analyzed data and provided critical input. JCM, MAC, and DCR analyzed data, provided critical input, and obtained financial support. NOD designed the study, analyzed data, provided critical input, and obtained financial support.

## Supplementary Material

Supplemental data

Supplemental data set 1

Supplemental video 1

## Figures and Tables

**Table 1:**
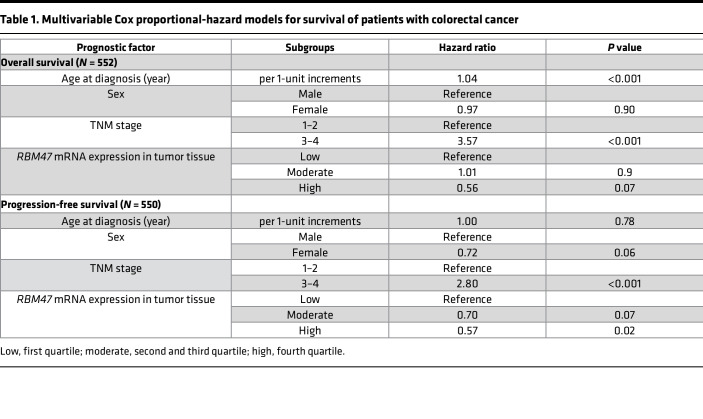
Multivariable Cox proportional-hazard models for survival of patients with colorectal cancer

## Version 1 04/04/2023

In-Press Preview

## Version 2 05/08/2023

Electronic publication
