# Supplementary material for: RBM47 regulates intestinal injury and tumorigenesis by modifying proliferation, oxidative response, and inflammatory pathways
Source: JCI Insight. 2023 May 8;8(9):e161118. doi: 10.1172/jci.insight.161118 (PMC10243830; doi:10.1172/jci.insight.161118)
Supplement: Supplemental data [file jciinsight-8-161118-s099.pdf]

SUPPLEMENTAL FIGURE LEGENDS

Supplemental Figure 1. Constitutive and inducible intestinal *Rbm47* deletion (*Rbm47-IKO*) alters morphology of intestinal epithelium in young (8-14 wk) mice. A. Left: *Rbm47* mRNA expression in scraped mucosa from different segments of small intestine and colon in young *Rbm47$^{f/f}$* and *Rbm47-IKO* mice (mean ± SE, unpaired t-test, n= 5 per genotype); *** *P*<.001, **** *P*<.0001. Right: RBM47 protein abundance in jejunal scraped mucosa from young *Rbm47$^{f/f}$* and *Rbm47-IKO* mice (each lane represents pooled protein extracts from two separate mice, N= 6 per genotype). B. Body weight in young *Rbm47$^{f/f}$* and *Rbm47-IKO* mice (mean ± SE, unpaired t-test, n= 27 *f/f*, 15 *IKO*). C. Abnormal villus incidence (left), villus length (middle), and villus width in different segments of small intestine in young *Rbm47$^{f/f}$* and *Rbm47^{IKO}* mice (mean ± SE, unpaired t-test, n=6/genotype, 30-40 villi with complete longitudinal cross-sectional view per mice); * *P*<.05, ** *P*<.01, *** *P*<.001. D. Immunochemical staining of RBM47 in sections of middle small intestine (top, scale bar: 100 μm and colon bottom scale bar: 50 μm) from young *Rbm47$^{f/f}$* and *Rbm47-IKO* mice. E. Representative H&E-stained sections of middle small intestine from young inducible *Rbm47-IKO* mice (scale bar: 50μm) with abnormal villi (red arrow-heads) and crypt fission (yellow arrow-heads). F. Crypt depth (left), villus width (middle), and villus length (right) in different segments of small intestine from young *Rbm47$^{f/f}$* and inducible *Rbm47-IKO* mice (mean ± SE, unpaired t-test, n=6/genotype, 30-40 crypts and villi with complete longitudinal cross-sectional view per mice); * *P*<.05, ** *P*<.01 *** *P*<.001, **** *P*<.0001.

Supplemental Figure 2. Constitutive intestinal *Rbm47* deletion (*Rbm47-IKO*) does not alter colonic mucus thickness or intestinal permeability. A. Top left: abundance of Alcian blue positive cells in colonic crypts from young *Rbm47$^{f/f}$* and *Rbm47^{IKO}* mice (mean ± SE, unpaired t-test, n=4/genotype, 40 crypts with complete longitudinal cross-sectional view per mice). Top right: colon mucus thickness in young *Rbm47$^{f/f}$* and *Rbm47^{IKO}* mice (mean ± SE, unpaired t-test,

n=5/genotype, 20 40x section per mice). Bottom: Periodic acid-Schiff-stained sections of colon from young *Rbm47*$^{f/f}$ and *Rbm47*^{IKO} mice (scale bar: 50 μm). B. Serum FITC labeled dextran (FD4) concentration 4 hours after FD4 gavage in young *Rbm47*$^{f/f}$ and *Rbm47*^{IKO} mice (mean ± SE, unpaired t-test, n=5/genotype).

Supplemental Figure 3. *Rbm47* expression in different intestinal epithelial cells of mouse. A. Left: Single cell RNA-seq survey of *Rbm47* mRNA expression in murine intestinal epithelial cells. Data derived from (17) and https://singlecell.broadinstitute.org (total 28273 cells from 6 mice). Right: Categorization of single cell scatter map based on small intestine anatomical regions (top, cell count: duodenum 3768, ileum 3927, jejunum 3970 cells) and different subtypes of small intestine villus and crypt epithelial cells (bottom, cell count: enterocyte 1041, enteroendocrine 324, EP (enterocyte progenitor) 1957, goblet 1559, Paneth 529, stem 3679, TA (transit amplifying) 2311, tuft 265 cells). B. Left: Three-dimensional exhibition of single cell RNA-seq survey for *Rbm47* mRNA expression in murine intestinal epithelial cells. Right: Categorization of single cell scatter map based on small intestine epithelial cells with different levels of maturation (cell count: enterocyte 1041, EP (enterocyte progenitor) 1957, stem (3679), and TA (transit amplifying) 2311). C. *Rbm47* mRNA expression in murine intestinal epithelial cells from different anatomical regions (n= 6 mice, cell count: duodenum 3768, ileum 3927, jejunum 3970 cells). D. *Rbm47* mRNA expression in different subtypes of murine intestinal epithelial cells (n= 6 mice, cell count: enterocyte 1041, enteroendocrine 324, EP (enterocyte progenitor) 1957, goblet 1559, Paneth 529, stem 3679, TA (transit amplifying) 2311, tuft 265 cells).

Supplemental Figure 4. Differentially expressed genes in small intestine from young (8-14 wk) *Rbm47-IKO* mice. A. Gene ontology molecular function (left) and biological process (right) enrichment in upregulated genes. B. Gene ontology molecular function (left) and biological

process (right) enrichment in downregulated genes. C. aligned features in the 3' untranslated region of intestinal RNAs whose expression was changed with *Rbm47* deletion.

Supplemental Figure 5. Constitutive intestinal *Rbm47* deletion shifts polysomal distribution of *Fndc5* mRNA. Relative *Fndc5* mRNA abundance upon fractionation of jejunal mucosa extracts from *Rbm47*$^{f/f}$ and *Rbm47-IKO* mice: monosome (fractions 1-6) and polysome (fractions 7-13). Data are representative of 3 separate fractionations.

Supplemental Figure 6. Constitutive intestinal *Rbm47* deletion (*Rbm47-IKO*) does not influence apoptosis or stromal total COX2-positive cell content of small intestine after 12 Gy whole-body irradiation. A. Left: TUNEL-stained sections of middle small intestine crypts from *Rbm47*$^{f/f}$ (top) and *Rbm47-IKO* (bottom) mice 6 hours after irradiation (scale bar: 50 μm). TUNEL-positive crypt cells are marked by yellow arrows. Right: TUNEL-positive cell abundance (%) per small intestine crypt from *Rbm47*$^{f/f}$ and *Rbm47-IKO* mice 6 hours after irradiation (mean ± SE, unpaired t-test, n=5/genotype, 20-30 crypts with complete longitudinal cross-sectional view per mice). B. total number of COX2-positive cells per 80x high power field of intestinal stroma from *Rbm47*$^{f/f}$ and *Rbm47-IKO* mice after irradiation (mean ± SE, unpaired t-test, n=5/genotype, 20-30 high power fields per mice).

Supplemental Figure 7. Constitutive intestinal *Rbm47* deletion (*Rbm47-IKO*) promotes spontaneous intestinal polyposis and dysplastic intestinal polyps in *Apc*$^{min/+}$ mice. A. number of aged *Rbm47*$^{f/f}$ and *Rbm47-IKO* mice with spontaneous polypogenesis in small intestine and colon. B. H&E-stained sections with increasing magnifications of polyps in small intestine from *Apc*$^{min/+}$ *Rbm47*$^{f/f}$ and *Apc*$^{min/+}$*Rbm47-IKO* mice (scale bar: Top 200 μm, bottom 100 μm). C. Rbm47-stained sections of small intestinal polyps from *Apc*$^{min/+}$ *Rbm47*$^{f/f}$ and *Apc*$^{min/+}$*Rbm47-IKO* mice (scale bar: 100 μm).

Supplemental Figure 8. Constitutive intestinal *Rbm47* deletion (*Rbm47-IKO*) mitigates colitis-associated tumorigenesis after azoxymethane (AOM) / DSS treatment. A. Extent of dysplastic lesions in middle (left) and distal colon (right) from *Rbm47*$^{f/f}$ and *Rbm47-IKO* mice after AOM-DSS treatment (chi-squared test, n= 10 *f/f* and 12 *IKO* mice). B. BrdU+ cell abundance (left) and representative BrdU-stained sections (right, scale bar= 50 µm) of dysplastic colonic lesions from AOM-DSS treated *Rbm47*$^{f/f}$ and *Rbm47-IKO* mice (mean ± SE, unpaired t-test, n=6/genotype, 20 40x field per mice); *P<.05.

Supplemental Figure 9. Constitutive intestinal *Rbm47* deletion modifies alternative splicing of *Zo1 (Tjp1)* in mice. A. rtPCR electrophoresis gel image illustrating mRNA abundance of total *Zo1*, the short isoform (*Zo1 -20*), and the long isoform (*Zo1 +20*) in small intestine from *Rbm47*$^{f/f}$ and *Rbm47-IKO* mice (each band represents pooled cDNA from two mice: n= 4/genotype). B. qPCR evaluation of *Zo1 +20*/total *Zo1* ratio in small intestine from young *Rbm47*$^{f/f}$ and *Rbm47-IKO* mice (left; mean ± SE, unpaired t-test, n=6 *f/f* and 7 *IKO*), small intestinal stem-cell derived enteroids from young *Rbm47*$^{f/f}$ and *Rbm47-IKO* mice (middle; mean ± SE, unpaired t-test, n= 5 *f/f* and 6 *IKO* mice), and uninvolved and paired polyp tissues from aged (one-year old) *Rbm47-IKO* mice intestine (right; mean ± SE, paired t-test, n=6); *P<.05, ***P<.001, ****P<.0001.

Supplemental Figure 1

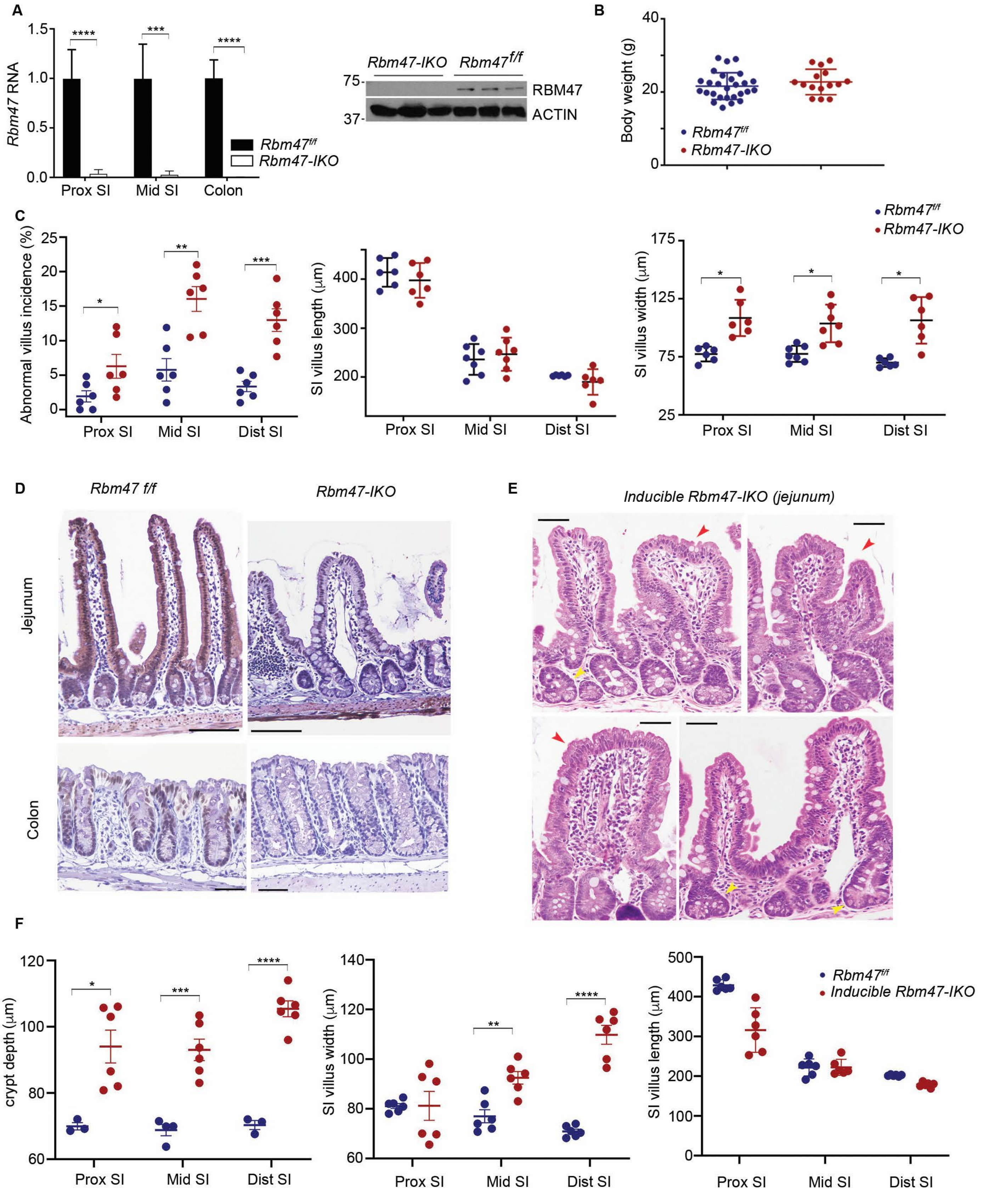

A

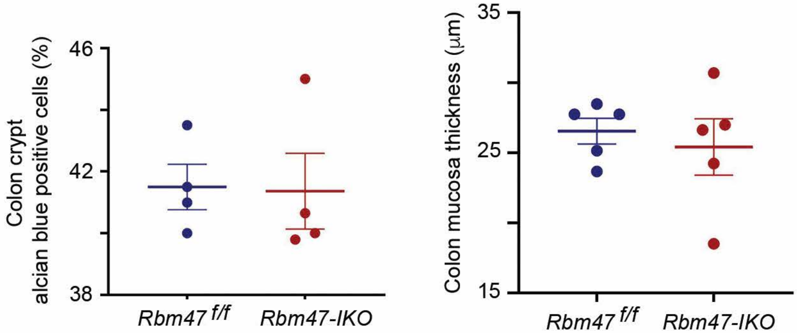

B

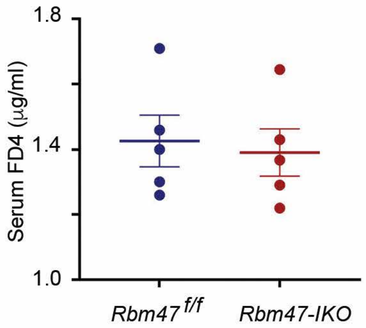

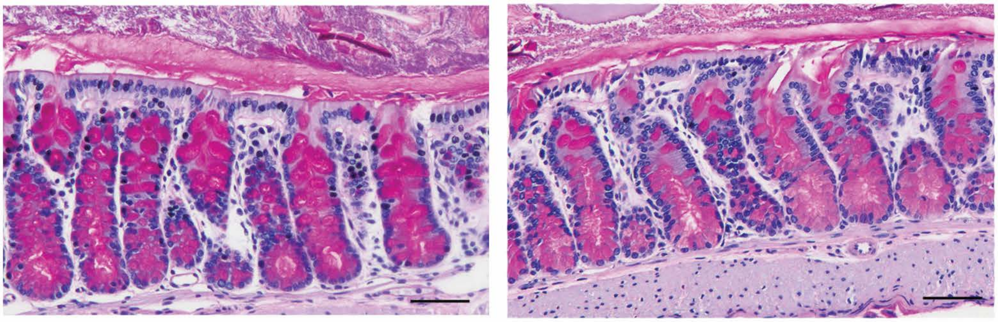

Rbm47$^{f/f}$ *Rbm47-IKO*

Supplemental Figure 3

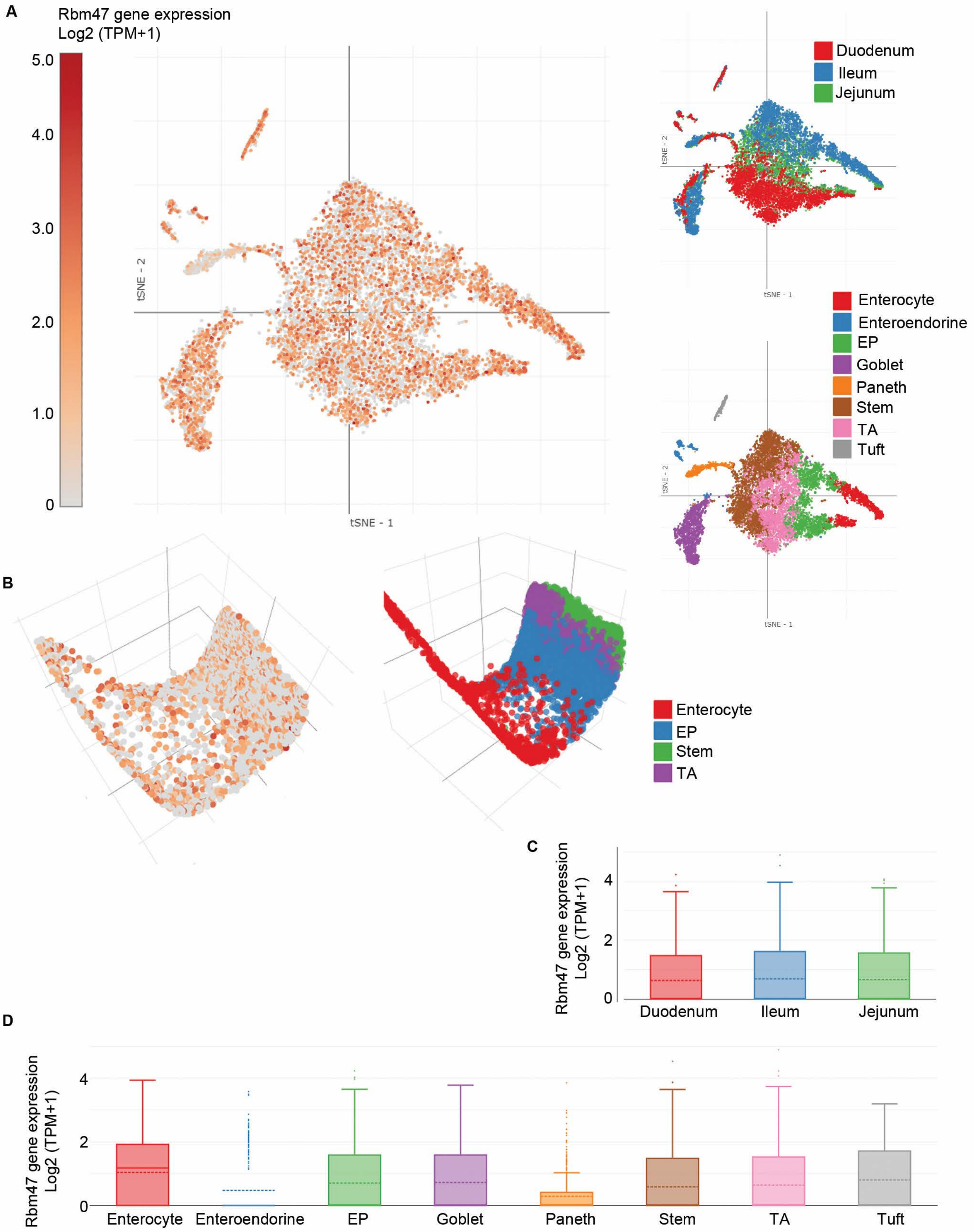

A

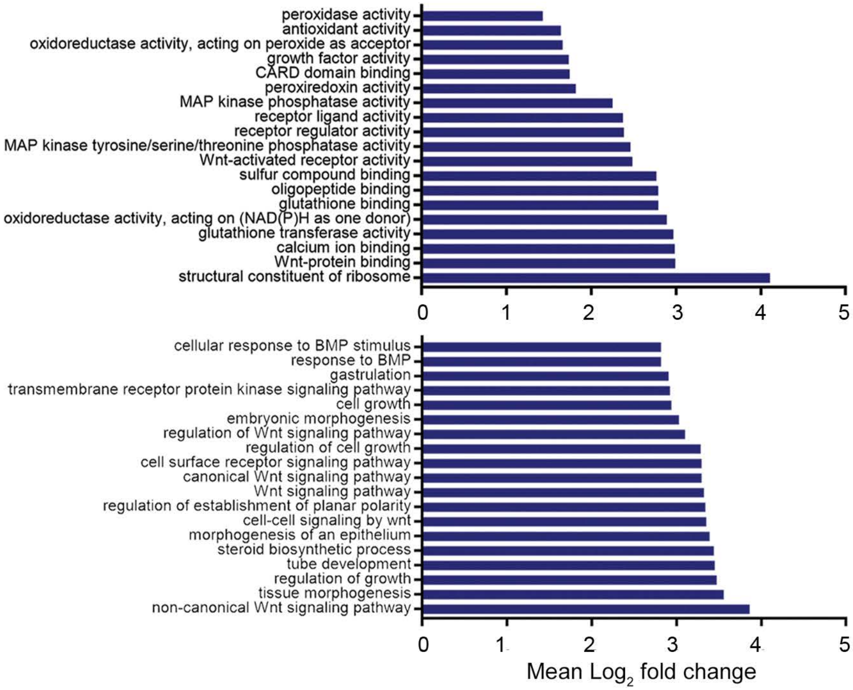

B

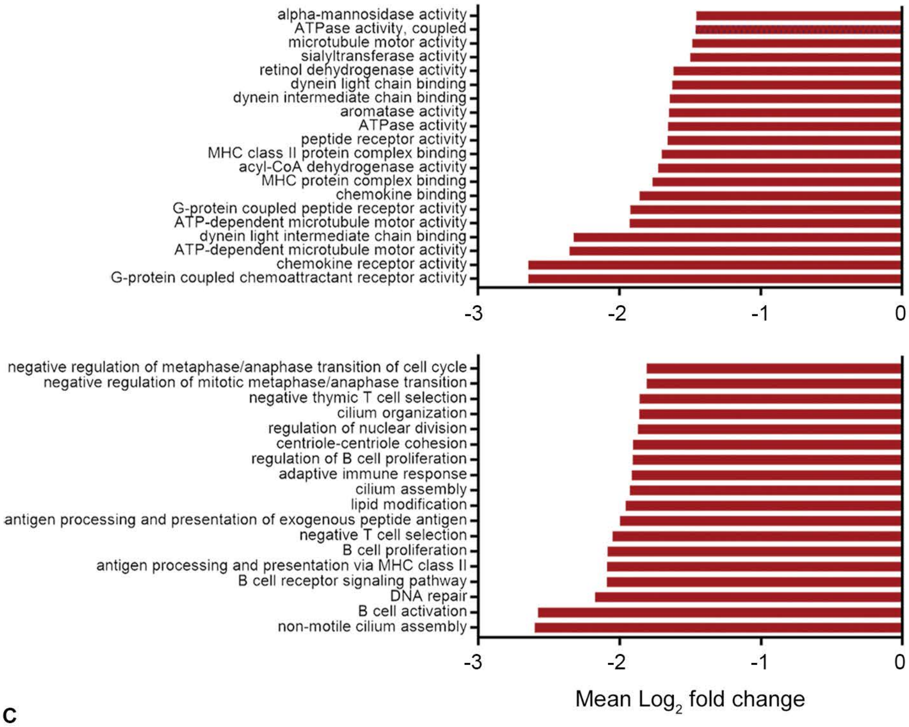

C

Gene	5U	≥6 U	AUUUA	AU%
Atoh1	2	1	2	59
Cdk2	2	1	2	56
Epop	0	0	0	47
Fndc5	1	0	0	50
Gsta3	0	0	1	63
Gsta4	0	0	0	57
Gstm1	0	0	0	44
HoxA3	3	1	4	63

Gene	5U	≥6 U	AUUUA	AU%
Hoxb13	0	0	0	49
Il33 Is1	1	0	3	59
Lgr5	4	1	7	61
Lrig1	0	0	2	55
Nqo1	5	4	1	59
Phlda1	1	1	2	60
Smoc2	0	0	2	54
Sod1	0	0	0	65

A

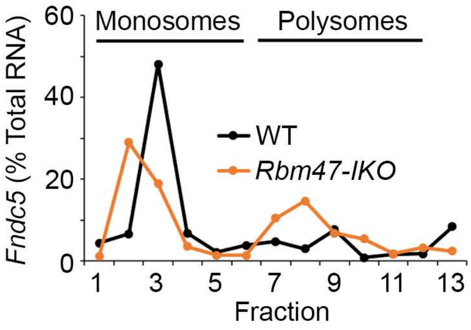
60
40
20
0
Monosomes
Polysomes
Fndc5 (% Total RNA)
WT
Rbm47-IKO
1
3
5
7
9
11
13
Fraction

A

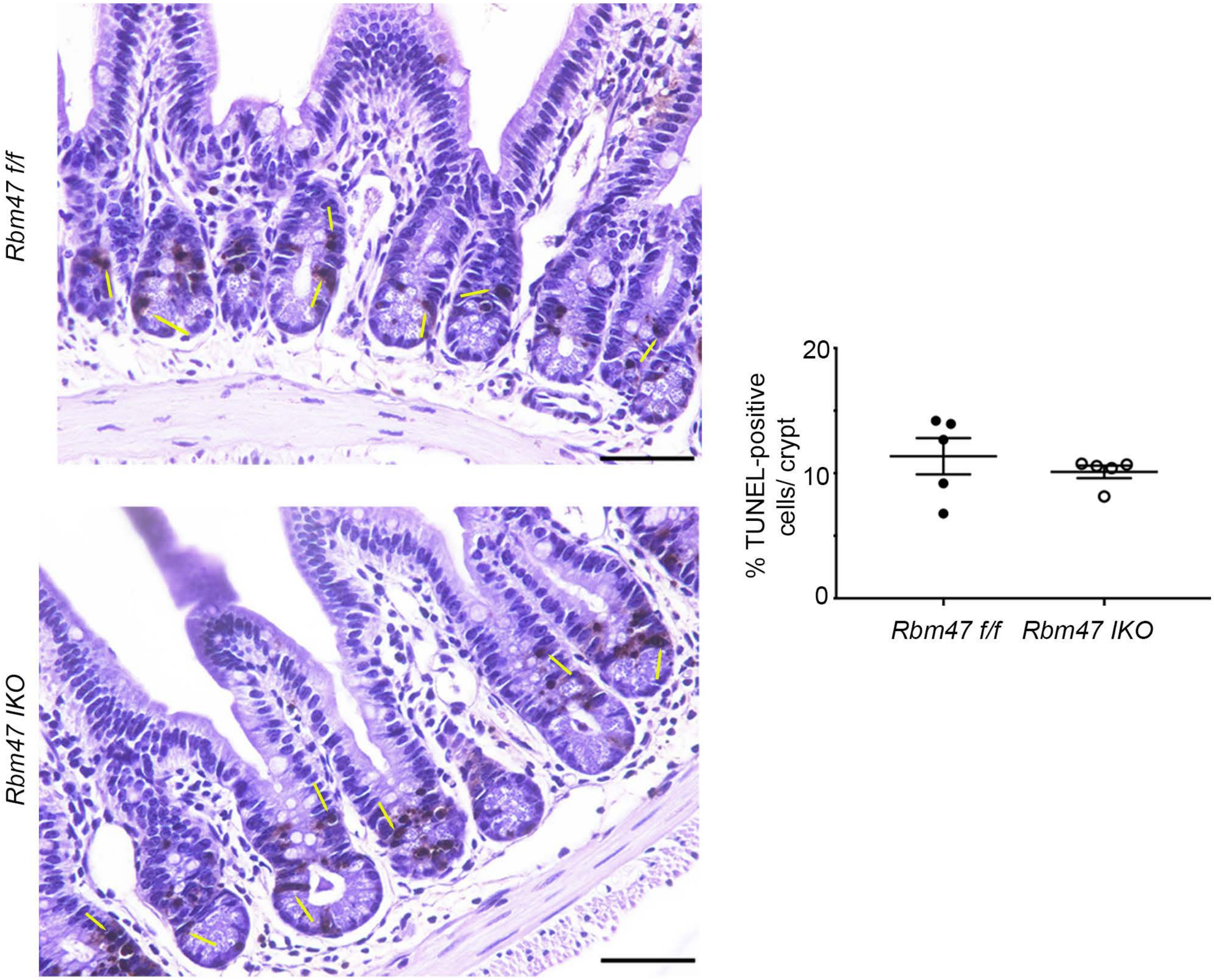
Rbm47 f/f
Rbm47 IKO
20
10
0
% TUNEL-positive cells/ crypt
Rbm47 f/f
Rbm47 IKO

B

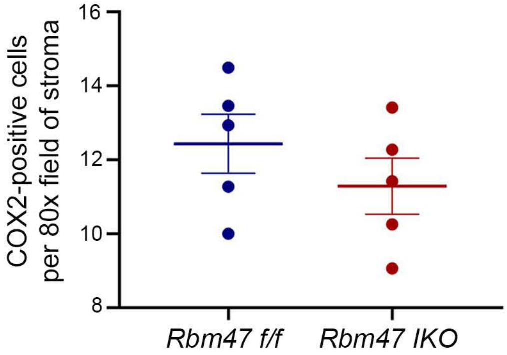
16
14
12
10
8
COX2-positive cells per 80x field of stroma
Rbm47 f/f
Rbm47 IKO

Supplemental Figure 7

A

Genotype	number of aged mice: Polyp	number of aged mice: no polyp
Rbm47 $^{f/f}$	1	12
Rbm47-IKO	11	1

B

Apc $^{min/+}$ *Rbm47* $^{f/f}$ *Apc* $^{min/+}$ *Rbm47 IKO*

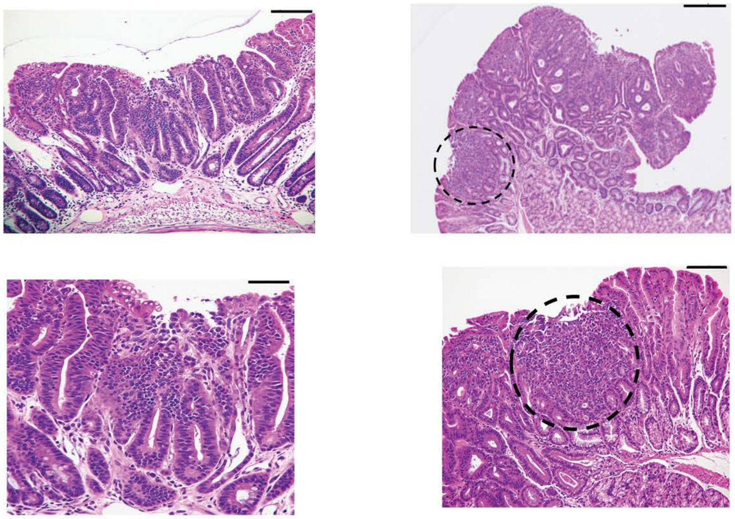

C

Apc $^{min/+}$ *Rbm47* $^{f/f}$ *Apc* $^{min/+}$ *Rbm47 IKO*

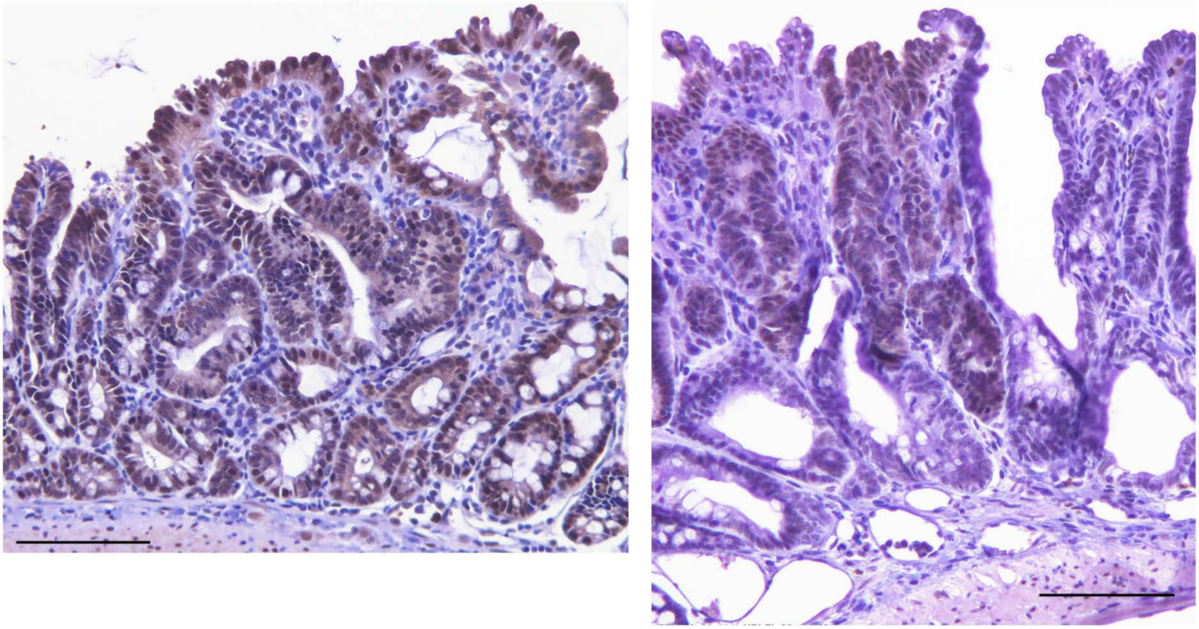

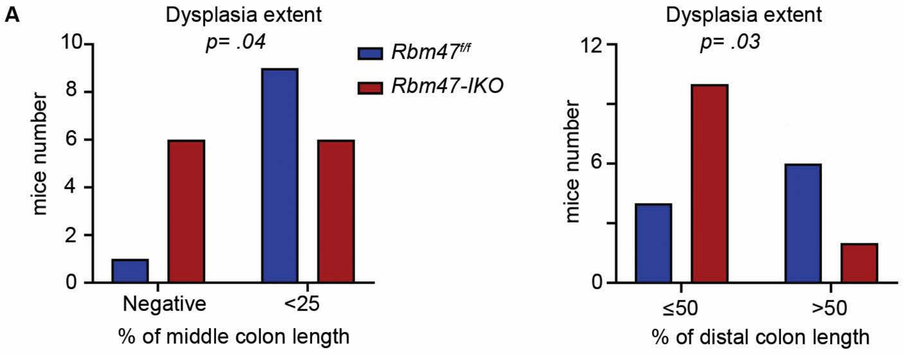
A
Dysplasia extent
p= .04
Rbm47f/f
Rbm47-IKO
mice number
10
8
6
4
2
0
Negative
<25
% of middle colon length
Dysplasia extent
p= .03
12
6
0
mice number
≤50
>50
% of distal colon length

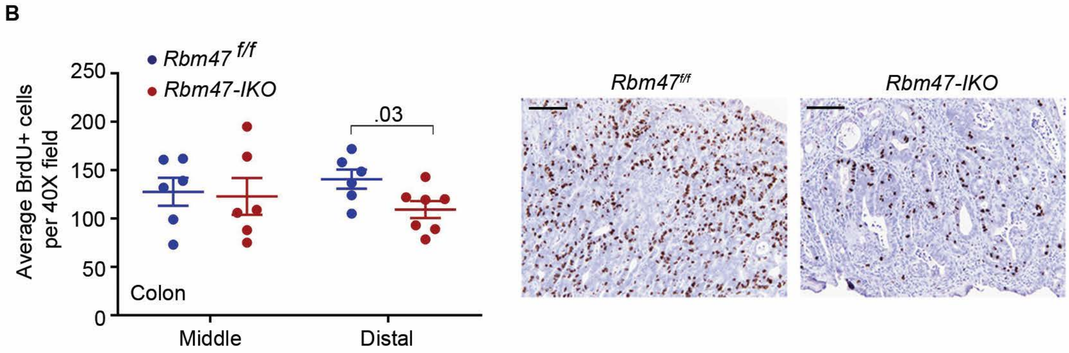
B
Rbm47 f/f
Rbm47-IKO
Average BrdU+ cells per 40X field
250
200
150
100
50
0
.03
Colon
Middle
Distal
Rbm47f/f
Rbm47-IKO

A

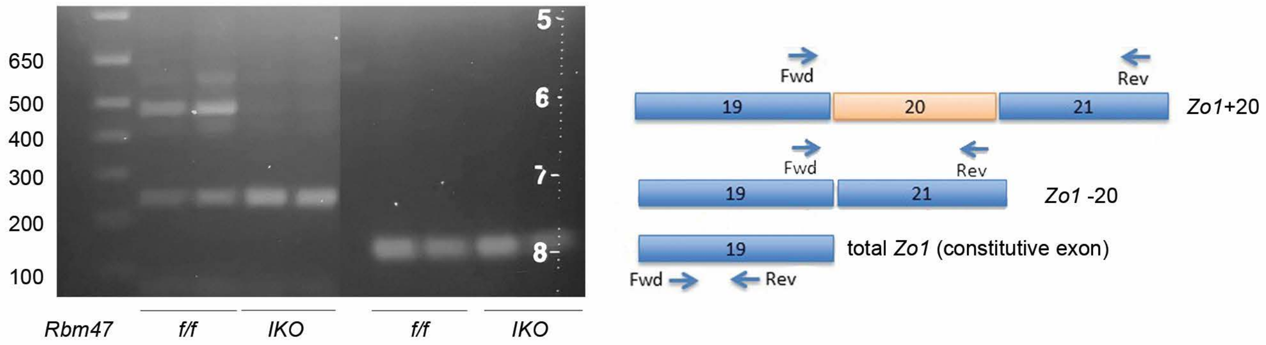
650
500
400
300
200
100
5
6
7
8
Rbm47
f/f
IKO
f/f
IKO
Fwd
Rev
19
20
21
Zo1+20
Fwd
Rev
19
21
Zo1 -20
19
total Zo1 (constitutive exon)
Fwd
Rev

B

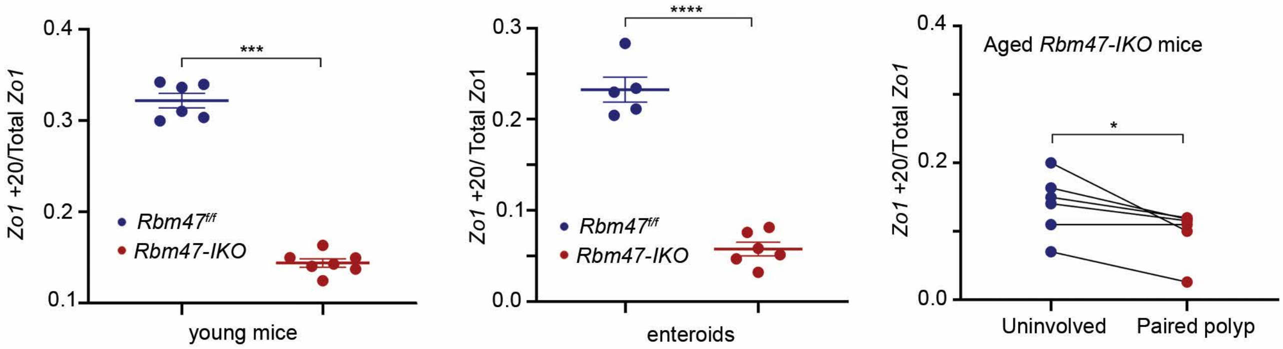
Zo1 +20/Total Zo1
0.4
0.3
0.2
0.1
***
Rbm47f/f
Rbm47-IKO
young mice
Zo1 +20/ Total Zo1
0.3
0.2
0.1
0.0
****
Rbm47f/f
Rbm47-IKO
enteroids
Zo1 +20/Total Zo1
0.4
0.2
0.0
Aged Rbm47-IKO mice
*
Uninvolved
Paired polyp

Supplemental Table 1. Disease activity scoring in mice treated with Dextran Sulfate Sodium.			
Body weight loss	Feces consistency	Fecal bleeding	Terminal outcome
0-1% (0)	Well-formed pellet: 0	Negative: 0	Death: 12
1-5% (1)	Loose stool:2	Blood-stained feces: 2	BWL >20%: 12
5-10% (2)	Frank Diarrhea: 4	Rectal bleeding: 4	
10-15% (3)			
>15% (4)			
